# PLEK2 promotes gallbladder cancer invasion and metastasis through EGFR/CCL2 pathway

**DOI:** 10.1186/s13046-019-1250-8

**Published:** 2019-06-10

**Authors:** Hui Shen, Min He, Ruirong Lin, Ming Zhan, Sunwang Xu, Xince Huang, Chu Xu, Wei Chen, Yanhua Yao, Man Mohan, Jian Wang

**Affiliations:** 1 Department of Biliary-Pancreatic Surgery, Renji Hospital, School of Medicine, Shanghai Jiao Tong University, 160 Pujian Road, Shanghai, 200127 China; 2 Department of Biochemistry and Molecular Cell Biology, Shanghai Key Laboratory of Tumor Microenvironment and Inflammation, Institutes of Medical Sciences, School of Medicine, Shanghai Jiao Tong University, Shanghai, 200025 China

**Keywords:** PLEK2, EGFR, CCL2, Metastasis, Gallbladder Cancer

## Abstract

**Background:**

Gallbladder cancer (GBC) is an extremely malignant tumor with a high mortality rate. Little is known about its invasion and metastasis mechanism so far.

**Methods:**

To identify the driver genes in GBC metastasis, we performed a mRNA microarray of metastatic GBC and paired non-tumor samples, and found PLEK2 was markedly upregulated in GBC tissues. Next, the expression of PLEK2 in GBC were examined in a larger cohort of patients by qRT-PCR, western blot and IHC staining. The clinicopathologic correlation of PLEK2 was determined by statistical analyses. The biological involvement of PLEK2 in GBC metastasis and the underlying mechanisms were investigated.

**Results:**

In this study, we found that PLEK2 had higher expression in GBC tumor tissues compared to non-cancerous adjacent tissues and cholecystolithiasis tissues. The clinicopathologic analyses showed PLEK2 expression was positively correlated with tumor TNM stage, distant metastasis and PLEK2 was an independent predictor of overall survival (OS) in GBC patients. The cellular function assays showed PLEK2 promoted GBC cells migration, invasion and liver metastasis in mouse model via the regulation of epithelial-mesenchymal transition (EMT) process. Our mass spectrum and co-immunoprecipitation (co-IP) assays demonstrated that PLEK2 could interact with the kinase domain of EGFR and suppress EGFR ubiquitination mediated by c-CBL, leading to constitutive activation of EGFR signaling. Furthermore, RNA-sequencing and qRT-PCR results demonstrated chemokine (C-C motif) ligand 2 (CCL2), a target gene downstream of PLEK2/EGFR signaling, mediated the motility-promoting function of PLEK2.

**Conclusions:**

On the basis of these collective data, we propose that PLEK2 promotes the invasion and metastasis of GBC by EGFR/CCL2 pathway and PLEK2 can serve as a potential therapeutic target for GBC treatment.

**Electronic supplementary material:**

The online version of this article (10.1186/s13046-019-1250-8) contains supplementary material, which is available to authorized users.

## Background

Gallbladder cancer (GBC) is the most common tumor in biliary tract disease. The median survival is less than 1 year and overall survival rate is about 17.8–21.7% [1, 2]. The prognosis of GBC is poor due to its high propensity to invasion and metastasis. Only 15–47% diagnosed GBC patients can be treated by surgery, while most of GBC patients miss the chance of surgery because of early metastasis [3, 4]. GBC can spread by lymph node metastasis, adjacent liver metastasis, vascular metastasis, trans-peritoneal metastasis and neural metastasis [5, 6]. However, molecular mechanisms involved in GBC metastasis are still poorly understood. Recent studies describe 20 to 59% of GBC shows the K-ras or ERBB mutations which may contribute to the malignant phenotype of GBC cells [7]. But the situation may be different in other GBC patients who don’t carry K-ras or ERBB mutations.

To identify the driver genes in GBC metastasis, we performed a mRNA microarray analysis of metastatic GBC and paired non-tumor samples and found several genes upregulated, including pleckstrin-2 (PLEK2). PLEK2 is a 353 amino acid protein with 2 pleckstrin homology (PH) domains and a disheveled–Egl-10–pleckstrin (DEP) domain [8]. Unlike Pleckstrin1 (PLEK1), which has homology to PLEK2 and restricted in immune cells, PLEK2 has been detected in various tissues [8]. PLEK2 can redistribute the actin within cells and cause more microvilli and large lamellipodia with ruffle formation, inducing the cell spreading. In addition, PLEK2 can interact with PI3K (phosphatidylinositol 3-kinase) lipid products like PI (3,4,5) P3 and PI (3,4) P2 [9, 10]. Upon the stimulation of the T-cell receptor α4β1, PLEK2 moves to the cell membrane with the help of its connection with PI3K lipid products [9, 10]. Recently, PLEK2 was found to play crucial roles in cancer metastasis and progression. Naume et al. found PLEK2 expression was correlated with luminal A type breast cancer cells disseminating to bone marrow and the disseminated tumor cell status predicted clinical outcome [11]. Meanwhile, a large whole blood-based transcriptome analysis identified PLEK2 expression was the strongest gene to distinguish CD45^−^ subsets melanoma patients from healthy people. Transcriptome profiling of PLEK2 expression in whole blood cells could be used as early detection of melanoma [12]. Altogether, the role of PLEK2 in tumor metastasis is being recognized gradually, but the clear mechanism of how it works is poorly understood. Moreover, to our knowledge, the role of PLEK2 in GBC has not been studied before.

Our protein–protein interaction analysis suggests that PLEK2 can interact with EGFR. Whether PLEK2 is involved in EGFR activation process is what we deal with in this study. EGFR is a 170 kDa receptor tyrosine kinase and widely expressed in numerous tumors [13]. EGFR aberrant activation has been taken as a leading cause of malignant transformation and cancer metastasis [14, 15]. Numerous studies show patients with high expression of EGFR tend to have short survival time. So far, the mechanism of EGFR aberrant activation has not been fully understood. The oncogenic activation of EGFR can be induced by various mechanism, such gene mutation, transcriptional overexpression, chromosomal translocation or defective degradation of EGFR [16, 17]. Lots of EGFR tyrosine kinase inhibitors (TKIs) and monoclonal antibodies have been used in clinical treatment [18, 19]. However, an increasing number of de novo and acquired drug resistance events have been identified [20]. Moreover, there are few studies concerning the role of EGFR in GBC progression.

In this study, we provided evidences both in vitro and in vivo that PLEK2 promoted GBC cells migration, invasion and liver metastasis via the regulation of epithelial-mesenchymal transition (EMT) process. Additionally, PLEK2 had higher expression in GBC tumor tissues compared to cholecystolithiasis tissues and high PLEK2 expression was positively correlated with liver metastasis and prognosis in GBC patients. Mechanical investigations verified that PLEK2 could combine with EGFR and suppress EGFR ubiquitination mediated by c-CBL, leading to constitutive activation of EGFR signaling. Furthermore, we found chemokine (C-C motif) ligand 2 (CCL2) mediated the motility-promoting function of PLEK2. In conclusion, our study demonstrated that PLEK2 promoted the invasion and metastasis of GBC by EGFR/CCL2 pathway.

## Materials and methods

### Patients

Tumor samples and paired normal samples were collected from GBC patients who underwent surgical resection and postoperative adjuvant chemotherapy. Total149 GBC tumor and 149 cholecystolithiasis samples were collected at the Department of Pathology, Renji Hospital, from January 2004 to February 2015. The 149 GBC tumor and 149 cholecystolithiasis tissues were used for tissue microarray, the 29 pairs of the fresh primary GBC tissues and paired non-tumorous tissues were used for qRT-PCR and the 14 pairs of tissues were used for western blotting assays. Medical records and Follow-up data were obtained from the questionnaires and patients’ medical records of the hospital. This project was approved by the Ethical Committee of Renji Hospital, Shang Hai Jiao Tong University School of Medicine.

### Immunohistochemistry (IHC)

Total 149 GBC tumor samples were stained with the PLEK2 (Proteintech), CCL2 (Proteintech) and EGFR (Santa Cruz) antibody. The staining was scored as the intensity of the positive staining (0 - negative, 1 - weak, 2 - moderate, 3 - strong) multiplied by the staining areas (0 = negative, 1 = 1 —9%, 2 = 10—39%, 3 = 40 —69%, and 4 = 70 —100%). These scores were independently determined by two pathologists.

### Cell lines, cell culture and construction of stable cell lines

Human GBC cell line GBC-SD and HEK 293 T cells were purchased from the Cell Bank of the Chinese Academy of Sciences. Human GBC cell line NOZ was obtained from Xinhua hospital (Shanghai, China). HEK 293 T and GBC-SD cells were cultivated in RPMI-1640 medium (GibcoBRL, Gaitherburg, MD, USA) supplemented with 10% fetal bovine serum (GIBCO) in an atmosphere consisting of 5% CO2 and 37 °C. Willian’s E medium was used in NOZ cells. The PLEK2 knockdown and overexpression cells were all constructed as stable cell lines. The Flag-tagged PLEK2 were cloned into into pCDH-CMV plasmid (System Biosciences, CA, USA). The PLKE2 shRNA was constructed by Shanghai GenePharma Medical Biotechnology Company. PCDH-flag-PLEK2 sense: 5′- TGCTCTAGAGCAATGGATTACAAGGA TGACGACGATAAGGAGGACGGCGTGCTCAAGGA -3′; PCDH-flag-PLEK2 antisense: 5′- CCGGAATTCCGGTCATGTTAGCTTTTTGATAGCTTCAATC − 3′. The sense sequence of PLEK2 shRNA was: TGCTGAGAGCTACAAAAAG; The sequence of the PLEK2 shRNA was the following: 5′-TGCTGTTGACAGTGAGC GCTTGCTGAGAGCTACAAAAAGATAGTGAAGCCACAGATGTATCTTTTTG TAGCTCTCAGCAAATGCCTACTGCCTCGGA-3′. The detailed methods of transfection and infection were previously described [21].

### Quantitative real time PCR (qRT-PCR)

Total RNA was isolated from GBC tissues and cell lines using Trizol RNA isolation reagent (Invitrogen) and reversely transcribed to cDNA with a cDNA Synthesis kit (Takara, Shiga, Japan). qRT-PCR was used to detected the gene expressions with SYBR Premix Ex Taq (Takara, Shiga, Japan). The primers were as follows:

PLEK2(F: 5′- TGGAGTTAAGTGGCACGGTG -3′; R: 5′- GAGCAGACACGAGTGAACCA − 3′); β-actin(F:5′- GGACTTCGAGCAAGAGATGG -3′;R:5′- AGCACTGTGTTGGCGTACAG-3′); EGFR(F: 5′- CTACAACCCCACCACGTACC-3′; R: 5′-CGCACTTCTTACACTTGCGG-3′); CCL2(F: 5′- AGCAGCAAGTGTCCCAAAGA -3′; R: 5′- TTGGGTTTGCTTGTCCAGGT-3′); ARHGDIB(F: 5′- ACTGGAGATCTGGAAGCCCT -3′; R: 5′- CCTGTAGGTGTGCTGAACGT-3′);

CNN3(F: 5′- ACGGGACTAGGAGGCATCTT-3′; R: 5′- GAGTTGTCCACCGGCTGTAA -3′); FGD4(F: 5′- TCCCTGGACTGGAATGATGC-3′; R: 5′- CCGAGCAGCTAGTTTGAGGA-3′); NEXN(F: 5′- AGAGAACGGAGGAGGAACGA-3′; R: 5′- TGTCCTCAATCTGTTCAGCCC -3′);

WIPF1(F: 5′- GCTTTGGGAGGAGGCTCAAT-3′; R: 5′- TGTTCTGAGGAGGAGGAGGG -3′); ARRB1(F: 5′- CTCATGTCGGACAAGCCCTT-3′; R: 5′- GGGCACTTGTACTGAGCTGT-3′); TWIST1(F: 5′- GTCCGCAGTCTTACGAGGAG -3′; R: 5′- GCCAGCTTGAGGGT CTGAAT -3′); SLUG(F: 5′- GCTGGCCAAACATAAGCAGC -3′; R: 5′- CCTTGAAGCAACCAGGGTCT -3′); ZEB1(F: 5′- ACTTTAGTTGCTCCCTGTGCA-3′; R: 5′- CGATTACACCCAGACTGCGT -3′);

ZEB2(F: 5′- CACACAAGCCAGGGACAGAT-3′; R: 5′-ACGTTTCTTGCAGTTTGGGC -3′); SNAIL(F: 5′- ACCACTATGCCGCGCTCTT-3′; R: 5′- GGTCGTAGGGCTGCTGGAA-3′).

### Coimmunoprecipitation

Cells were transfected with the indicated constructs for 48 h and then cells were dissolved in IP lysis buffer (Thermo Fisher, Inc) with protease inhibitor cocktail (Sigma) and PMSF (Sigma) for 1 h at 4 °C. After centrifugation at full speed for 20 min, cleared supernatant were gently rotated with antibodies and protein A beads (Invitrogen) for 4 h at 4 °C. Then beads were washed four times with IP lysis buffer. The beeds were eluted in 1X SDS buffer. Primary antibodies were as followings: anti-PLEK2 (Proteintech), anti-EGFR (Cell Signal Technology), anti-IgG (Abcam), and anti-Flag (Sigma).

### Mass spectrometry

The analyses were performed in an HPLC system (Easy-nLC1000, Thermo Fisher Scientific, USA) and mass spectrometer (Orbitrap Elite, Thermo Fisher Scientific, USA). A 15-cm dish of Flag-PLEK2 overexpression NOZ cells and control cells were collected. The sample were prepared as described in the method of coimmunoprecipitation. Eluates were subjected to western blot and then stained by coomassie brilliant blue.

### Cell migration and invasion assays

GBC cells were performed transwell assay. For migration assay, GBC cells were seeded into the 24-well transwell chamber at a density of 4 × **10**^**4**^ cells in 100 μl serum-free medium with 8-μm pore size polycarbonate membrane (Corning, NY, USA). 600ul medium containing 10% FBS was added to the lower chamber. 4% paraformaldehyde was used to fix the migrated cells and coomassie brilliant blue was used to stain the cells after 16 h. For invasion assay, 8 × 10^4^ cells were maintained in the matrigel (BD, NY, USA) coated chamber for 48 h.Three independent experiments were carried out.

### Immunofluorescence (IF)

2× 10^4^ GBC-SD cells were plated in 24-well plates covered with sterile coverslips. After12 hours, cells were starved 16 h prior to 50 ng/ml EGF incubation with 10 min. In the control group, cells were not treated with EGF. Then the primary and matched secondary antibody diluted in PBS containing 2% BSA were used to stain PLEK2. Next, we washed the cells with PBS three times and stained cell nuclei with 4, 6-diamidino-2-phenylindole (DAPI). Three independent experiments were carried out.

### Xenograft studies in nude mice

4 × 10^6^ NOZ cells **diluted in 100** μl **PBS** were inoculated subcutaneously into the right lower regions of 4-week-old male nude mice. The mice were sacrificed after 7 weeks from the inoculation. The subcutaneous xenografts and livers were dissected and made into sections for haematoxylin and eosin (H&E) staining. All procedures were performed in accordance with the regulations of Renji Hospital, School of Medicine, Shanghai Jiao Tong University. All the procedures were according to the regulations of the RenJi Hospital of Shanghai Jiao Tong University.

### Statistical analysis

Data are expressed as mean ± SEM. An unpaired two-tailed Student’s t test and Pearson’s Χ2-test was used to analyze the variance of each experimental group. Kaplan–Meier method and log-rank test were used to estimate the survival probabilities. Cox proportional hazard regression model was performed in univariate and multivariate analysis. *P* < 0.05 was considered statistically significant.

## Results

### PLEK2 was up-regulated in gallbladder cancer and correlated with poor prognosis

**Fig. 1:**
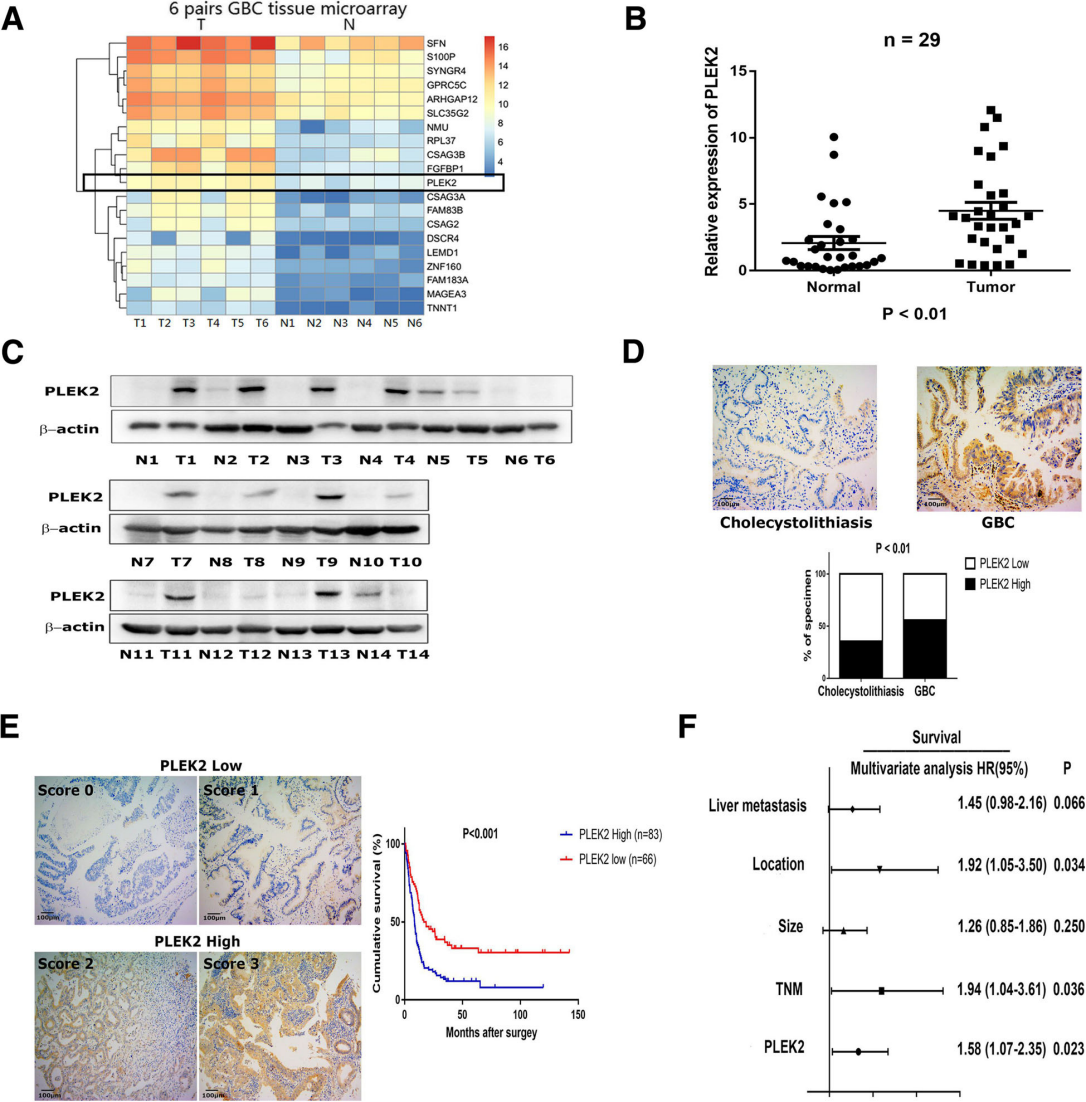
PLEK2 was up-regulated in gallbladder cancer and correlated with overall survival. **a** Heatmap results of a mRNA microarray consisted of six pairs of metastatic GBC and non-tumor samples. **b**, **c** The relative expression levels of PLEK2 in GBC tumor tissues (Tumor or T) and matched normal tissues (Non-Tumor or N) were detected by qRT-PCR (**b**) and western blot (**c**). **d** Representative IHC staining images of PLEK2 in cholecystolithiasis and GBC tissues, and quantification of PLEK2 expression according to IHC scores. **e** Representative IHC staining images of different scores calculated by intensity and percentage of stained cells and overall survival for the low and high PLEK2 expression groups. **f** Multivariate analysis of the factors associated with overall survival of GBC patients. All experiments were repeated at least three times, and data were analyzed using student’s t-test. *P < 0.05. Error bars indicate SEM

To identify the driver genes in GBC metastasis, we made a mRNA microarray consisted of six pairs of metastatic GBC and non-tumor samples (Fig. 1a). Analysis the gene expression differences and its distribution in human cancer cells by bioinformatics data (http://www.broadinstitute.org), we found that PLEK2, one of the most upregulated genes in GBC compared with paired non-tumor tissues, had a relative high expression in biliary tract cancer (Additional file 1: Figure S1A). qRT-PCR data of 29 pairs of GBC and non-cancerous adjacent tissues verified PLEK2 mRNA level was up-regulated in GBC compared with non-cancerous adjacent tissues (Fig. 1b). Additionally, we examined PLEK2 protein level in 14 pairs of GBC and non-tumor tissues using western blot analysis which showed most GBC tissues had higher PLEK2 protein level than the normal control (Fig. 1c). Similar results were also achieved in gallbladder tissues microarrays including 149 GBC and 149 cholecystolithiasis tissues by IHC analysis. As shown in Fig. 1d, PLEK2 expression was higher in GBC tissues than that in cholecystolithiasis tissues (*P* < 0.05). Altogether, these results suggested PLEK2 expression was elevated in GBC tissues. Besides, higher levels of the PLEK2 protein was found to positively correlate with TNM stage and liver metastasis (Table 1).

**Table 1:** Correlation of PLEK2 expression with the clinicopathological features of GBC

	PLEK2(High)		PLEK2 (Low)		*P* value
	*N* = 83	%	*N* = 66	%	
Sexual					0.623
Male	27	0.181	19	0.128	
Female	56	0.376	47	0.315	
Age (years)					0.271
≤ 65	39	0.262	37	0.248	
> 65	44	0.295	29	0.195	
Tumor size (cm)					0.256
≤ 3	35	0.235	34	0.228	
> 3	48	0.322	32	0.215	
T					0.010*
1–2	9	0.060	18	0.121	
3–4	74	0.497	48	0.322	
N					0.195
0	48	0.322	45	0.302	
1–2	35	0.235	21	0.141	
M					0.841
No	82	0.550	64	0.430	
Yes	1	0.007	2	0.013	
TNM stage					0.010*
I-II	9	0.060	18	0.121	
III-IV	74	0.497	48	0.322	
Tumor location					0.336
Body or bottom	73	0.490	62	0.416	
Neck or duct	10	0.067	4	0.027	
Liver metastasis					0.014*
No	44	0.295	48	0.322	
Yes	39	0.262	18	0.121	

*P < 0.05 was considered statistically significant

χ2 test was performed

We next sought to identify the clinicopathologic significance of PLEK2 in GBC, we investigated the relationship between PLEK2 expression and overall survival. Then we classified the GBC tissues into PLEK2 high and PLEK2 low groups according to PLEK2 expression level. The results showed PLEK2 high group had a significantly shorter overall survival compared with PLEK2 low group (HR:2.05, 95%CI:1.43–2.94, *P* < 0.001, Fig. 1e). Moreover, PLEK2 can be an independent factor for prognosis by multivariate analysis (Fig. 1f). All these data suggest PLEK2 expression was elevated in GBC and might promote the progression of GBC by enhancing the motility of GBC cells.

### PLEK2 promoted the migration, invasion and metastasis of GBC cells

**Fig. 2:**
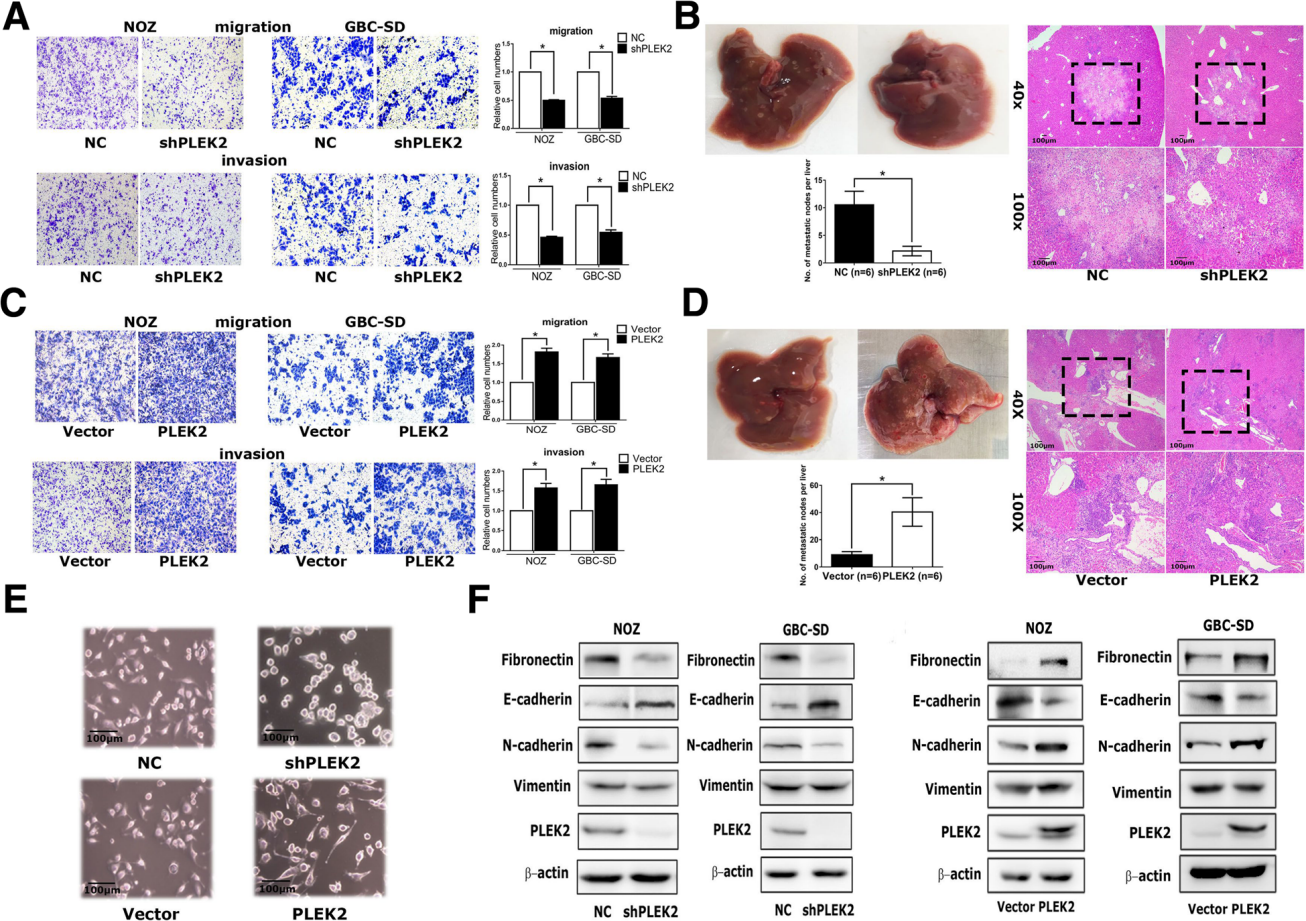
PLEK2 promoted the migration, invasion and metastasis of GBC cells. **a**, **c** Migration and invasion ability of NOZ and GBC-SD cells with stable PLEK2 knockdown (**a**) and PLEK2 overexpression (**c**) were measured by transwell migration and matrigel invasion assays. **b**, **d** Represent images of mouse livers of NOZ cells with stable PLEK2 knockdown (**b**) and PLEK2 overexpression (**d**) are shown and the numbers of metastatic nodes per liver were measured. **e** Morphological change of NOZ cells upon PLEK2 knockdown or overexpression. **f** Expression of epithelial marker E-cadherin, mesenchymal markers Fibronectin, Vimentin and N-cadherin were detected by western blot. All experiments were repeated at least three times, and data were analyzed using student’s t-test. * *P* < 0.05. Error bars indicate SEM

To investigate the causal role of PLEK2 in GBC progression, we constructed PLEK2 down-regulation NOZ and GBC-SD cells (NOZ-shPLEK2, GBC-SD-shPLEK2, respectively), also PLEK2 overexpression NOZ and GBC-SD cells (NOZ-PLEK2, GBC-SD-PLEK2, respectively) (Additional file 2: Figure S2A). Cell proliferation assay showed no difference between PLEK2 knockdown and control cells (Additional file 3: Figure S2B). Meanwhile, transwell migration assay indicated that PLEK2 knockdown or overexpression significantly inhibited or promoted cell migration in corresponding GBC cells, respectively. Similar with the migration assay, transwell invasion assay also showed the same results (Fig. 2a, c). Therefore, these in vitro studies indicated that PLEK2 promoted GBC cells migration and invasion. In addition, we investigated whether PLEK2 could promote GBC tumor metastasis in xenograft models. In vivo studies showed PLEK2 knockdown exhibited fewer liver metastatic foci whereas PLEK2 overexpression displayed more liver metastatic foci compared to the control group (Fig. 2b, d, Additional file 9: Figuer S5).

**Fig. 3:**
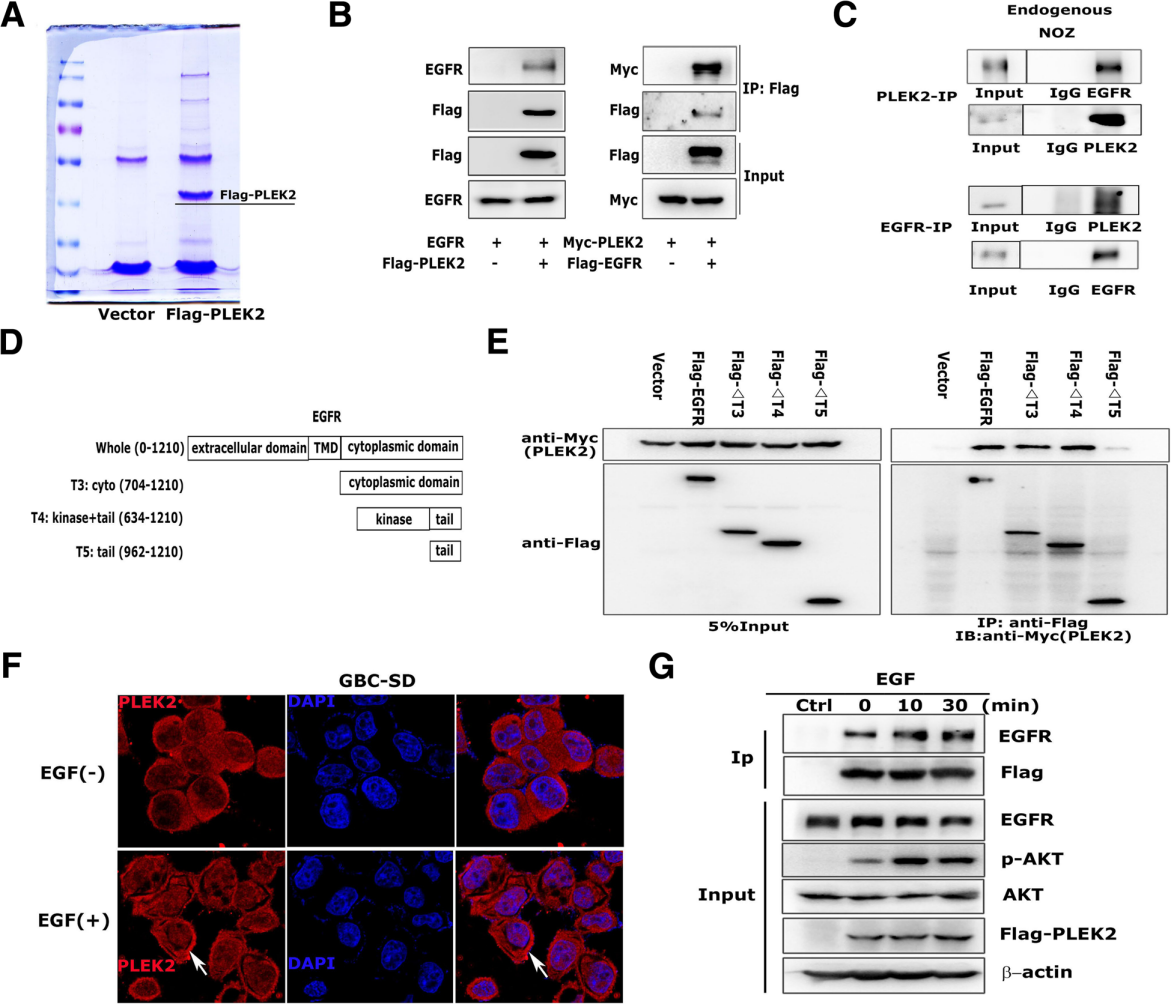
PLEK2 interacted with EGFR. **a** Total cell lysates extract from FLAG-PLEK2 stably expressed cells were subjected to affinity purification and mass spectrometry analysis of PLEK2-associated proteins were performed. **b** Interaction between exogenous PLEK2 and EGFR by Co-IP analyses in 293 T cells. **c** Interaction between endogenous PLEK2 and EGFR by Co-IP analyses in NOZ cells. **d**, **e** Mapping of the binding site of PLEK2/EGFR by Co-IP analyses. **f** Immunofluorescence assays in GBC-SD cells. The localization of PLEK2 was detected by confocal laser scanning microscopy as indicated. **g** Co-IP of overexpressed Flag-PLEK2 and EGFR in 293 T cells treated with 50 ng/mL EGF at indicated times

Given some previous studies have shown the involvement of PLEK2 in actin remodeling, we detected the morphological change following PLEK2 knockdown or overexpression. Consistent with previous studies, PLEK2 knockdown cells displayed small and round shape whereas PLEK2 overexpression cells exhibited spindle-like shape compared to control cells (Fig. 2e). The change in the cell morphology might facilitate their motility. As EMT process plays indispensable role in tumor metastasis, we investigated whether the function of PLEK2 in cell spreading promoted EMT process. As shown in Fig. 3f, PLEK2 knockdown suppressed Fibronectin and N-cadherin, whereas enhanced E-cadherin expression. On the contrary, PLEK2 overexpression enhanced Fibronectin and N-cadherin, whereas suppressed E-cadherin expression. Moreover, qRT-PCR got similar results in the mRNA level (Additional file 4: Figure S2C).

### PLEK2 interacted with EGFR

To further investigate the underlying mechanism by which PLEK2 promoted GBC migration and metastasis, we performed immunoprecipitation assay followed by mass spectrometry to identify PLEK2 interacting proteins (Fig. 3a). We found EGFR as one of the potential PLEK2 interacting protein from the identified list of proteins. The following co-IP assay verified the correlation between PLEK2 and EGFR (Fig. 3b). Immunoprecipitation of endogenous PLEK2 detected the presence of EGFR and the reciprocal co-IP also confirmed the correlation between PLEK2 and EGFR (Fig. 3c). EGFR has four functional domains: extracellular ligand-binding domain, transmembrane domain, and C-terminal regular domain [22]. To define the PLEK2 binding site, we constructed different truncates of EGFR and performed co-IP with PLEK2 (Fig. 3d). The results showed PLEK2 bind with the intracellular tyrosine kinase domain of EGFR (Fig. 3e).

As EGFR is a transmembrane receptor tyrosine kinase, the localization and kinetics of EGFR have pivotal impacts on its function and signaling. A number of different ligands, including EGF-like molecules, activate EGFR by binding to the extracellular domain. After EGF stimulation, EGFR signaling cascades can be transduced. As shown in Fig. 3f, PLEK2 migrated from the cytoplasm to the cell membrane and formed a strong IF staining in GBC cells membrane following EGF treatment. We examined whether the binding between PLEK2 and EGFR changed after EGF stimulation. Co-IP results showed more PLEK2 protein bound to EGFR after treating with EGF 10 or 30 min (Fig. 3g).

### PLEK2 suppressed EGFR degradation

**Fig. 4:**
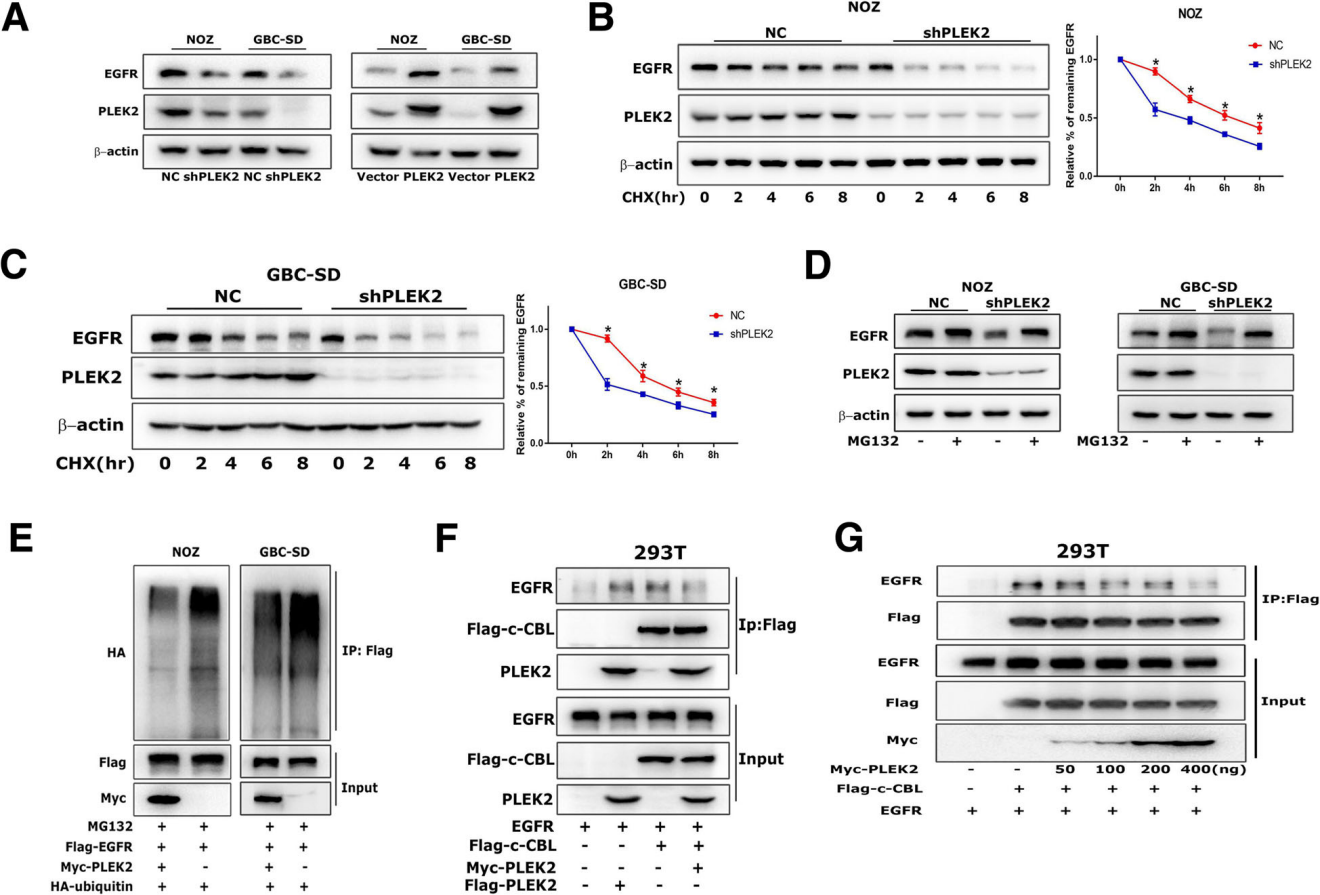
PLEK2 suppressed EGFR degradation. **a** Protein levels of EGFR in PLEK2 knockdown and overexpression cells were detected by western blot. **b**, **c** Alterations of EGFR degradation in PLEK2 knockdown cells in response to 100 nM CHX treatment for the indicated time were detected by western blot. **d** EGFR expression in PLEK2 knockdown cells in response to 10 μM MG132 treatment for 6 h were detected by western blot. **e** The ubiquitination level of EGFR with increasing ectopic PLEK2 expression were detected by co-IP. **f** Co-IP of overexpressed PLEK2, c-CBL and EGFR in 293 T cells. **g** Co-IP of Flag-c-CBL and EGFR, together with increasing amount of Myc-PLEK2 in 293 T cells. All experiments were repeated at least three times, and data were analyzed using student’s t-test. * *P* < 0.05. Error bars indicate SEM

PLEK2 knockdown significantly reduced EGFR protein level and PLEK2 overexpression markedly increased EGFR protein level by western blot analysis (Fig. 4a). In addition, the IF results confirmed that PLEK2 overexpression increased EGFR expression (Additional file 5: Figure S4A). After protein synthesis inhibitor CHX treatment for different time, EGFR expression was reduced immediately in PLEK2 knockdown cells compared to control cells (Fig. 4b, c), clearly indicating that knockdown of PLEK2 reduced the half-life of EGFR. Given that PLEK2 altered only the protein but not the mRNA levels of EGFR (Additional file 6: Figure S4B), we hypothesized that PLEK2 mainly regulated EGFR protein stability, not EGFR synthesis or transcription. Previous studies have demonstrated that EGFR protein degradation involved both protein ubiquitination mediated proteasome degradation and lysosome mediated degradation. We treated GBC cells with proteasome inhibitor MG132 and lysosome inhibitor chloroquine, separately. Western blot analysis revealed that PLEK2 mediated EGFR downregulation could be rescued by MG132 (Fig. 4d), but not by chloroquine (Additional file 7: Figure S4C). Furthermore, we performed ubiquitination assays to explore whether PLEK2 was involved in the regulation of EGFR ubiquitination. The levels of ubiquitylated EGFR were detected and were found to be increased in PLEK2 knockdown cells (Fig. 4e).

To clarify the underlying mechanism by which PLEK2 regulated EGFR ubiquitination, we analyzed the ubiquitin ligases of EGFR and found c-CBL was involved in the biological function of PLEK2. We co-expressed c-CBL, PLEK2 and EGFR in 293 T cells and performed co-IP assay with Flag antibody. Interestingly, we observed that PLEK2 inhibited the interaction between EGFR and c-CBL (Fig. 4f). Further, we co-expressed Flag-c-CBL and EGFR, together with increasing amount of Myc-PLEK2 in 293 T cells, and performed co-IP assay with Flag antibody. Consistence with the previous observation, increasing the amount of PLEK2 gradually inhibited the binding between EGFR and c-CBL (Fig. 4g). In addition, c-CBL could partially resist the increase in EGFR levels resulting from PLEK2 overexpression (Additional file 8: Figure S4D). All these results suggested that PLEK2 suppressed EGFR degradation through the competitive inhibition of the interaction between EGFR and c-CBL.

### Oncogenic effects of PLEK2 depended on EGFR pathways

**Fig. 5:**
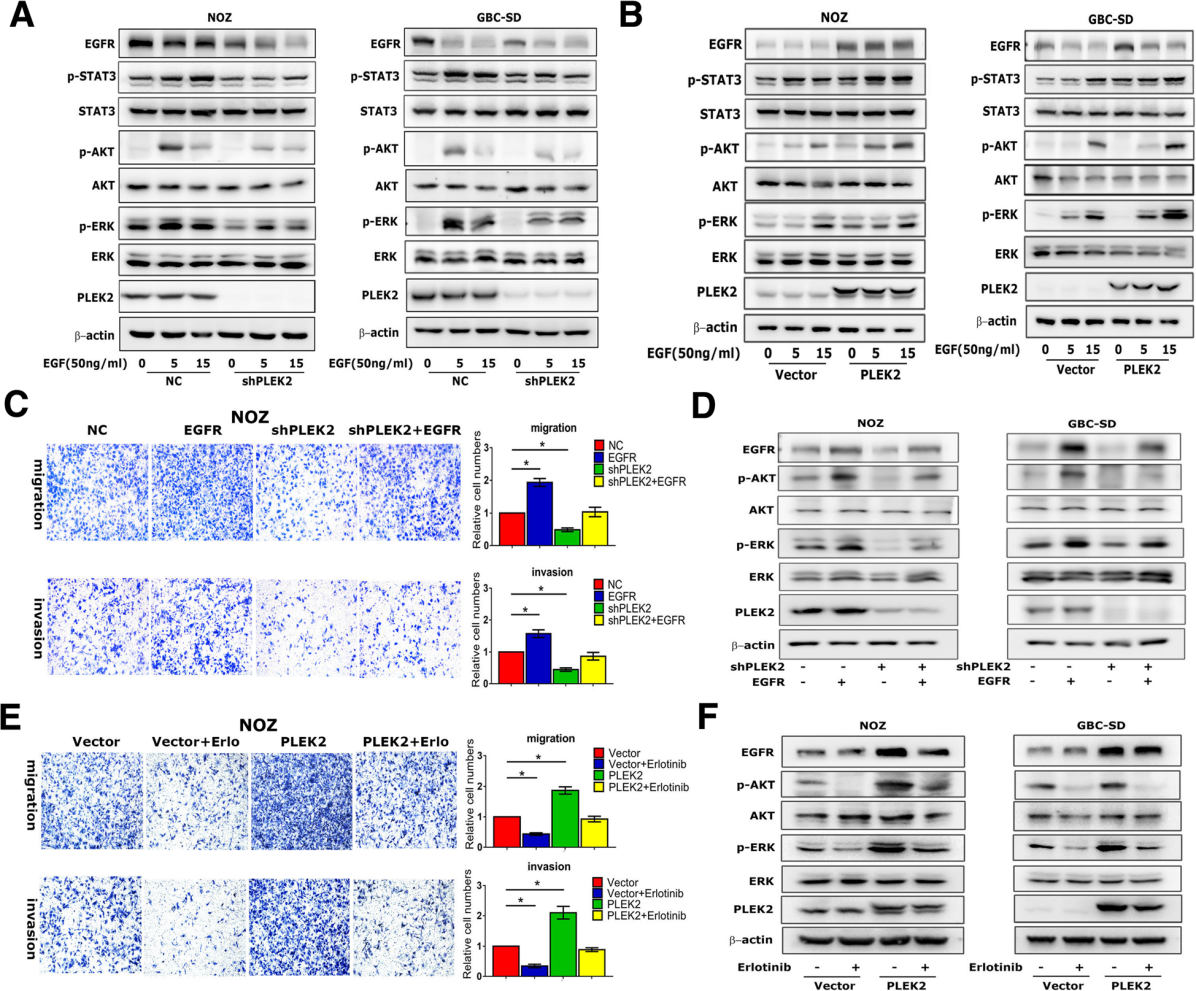
Oncogenic effects of PLEK2 depended on EGFR pathways. **a**, **b** Protein levels of EGFR downstream signaling in indicated GBC cell lines with either PLEK2 knockdown or overexpression were detected by western blot. **c** Migration and invasion ability of PLEK2 knockdown NOZ cells with increasing ectopic EGFR expression were measured. **d** Protein levels of EGFR downstream signaling in PLEK2 knockdown cells with increasing ectopic EGFR expression were detected by western blot. **e** Migration and invasion ability of PLEK2 overexpression NOZ cells with EGFR inhibitor erlotinib treatment were measured. **f** Protein levels of EGFR downstream signaling in PLEK2 overexpression cells with 1 mmol/L erlotinib treatment for 12 h were detected. All experiments were repeated at least three times, and data were analyzed using student’s t-test. * *P* < 0.05. Error bars indicate SEM

Given that PLEK2 suppressed the expression of EGFR, we wanted to determine whether PLEK2 regulated EGFR downstream signaling pathways. PI3K/AKT, MAPK/ERK and JAK/STAT were three main signaling pathways modulated by EGFR [23]. Western blot analysis showed PLEK2 knockdown reduced p-AKT, p-ERK and p-STAT3, while PLEK2 overexpression increased p-AKT, p-ERK and p-STAT3 expression (Fig. 5a, b). Furthermore, we increased EGFR levels in PLEK2 knockdown cells and checked whether it could compensate for loss of PLEK2. We found that increasing ectopic expression of EGFR in PLEK2 knockdown cells led to increased NOZ cell migration and invasion (Fig. 5c). The EGFR inhibitor erlotinib reduced the migration and invasion of PLEK2 overexpression cells (Fig. 5e). These results suggested that PLEK2 promoted GBC cell motility via EGFR signaling pathway. In addition, we also found that EGFR ectopic expression increased p-AKT and p-ERK expression in PLEK2 knockdown cells, while erlotinib treatment decreased p-AKT and p-ERK expression in PLEK2 overexpression cells (Fig. 5d, f). Thus, above results suggested that oncogenic effects of PLEK2 depended on EGFR downstream signaling pathways.

### CCL2 was a target gene downstream of PLEK2/EGFR signaling

**Fig. 6:**
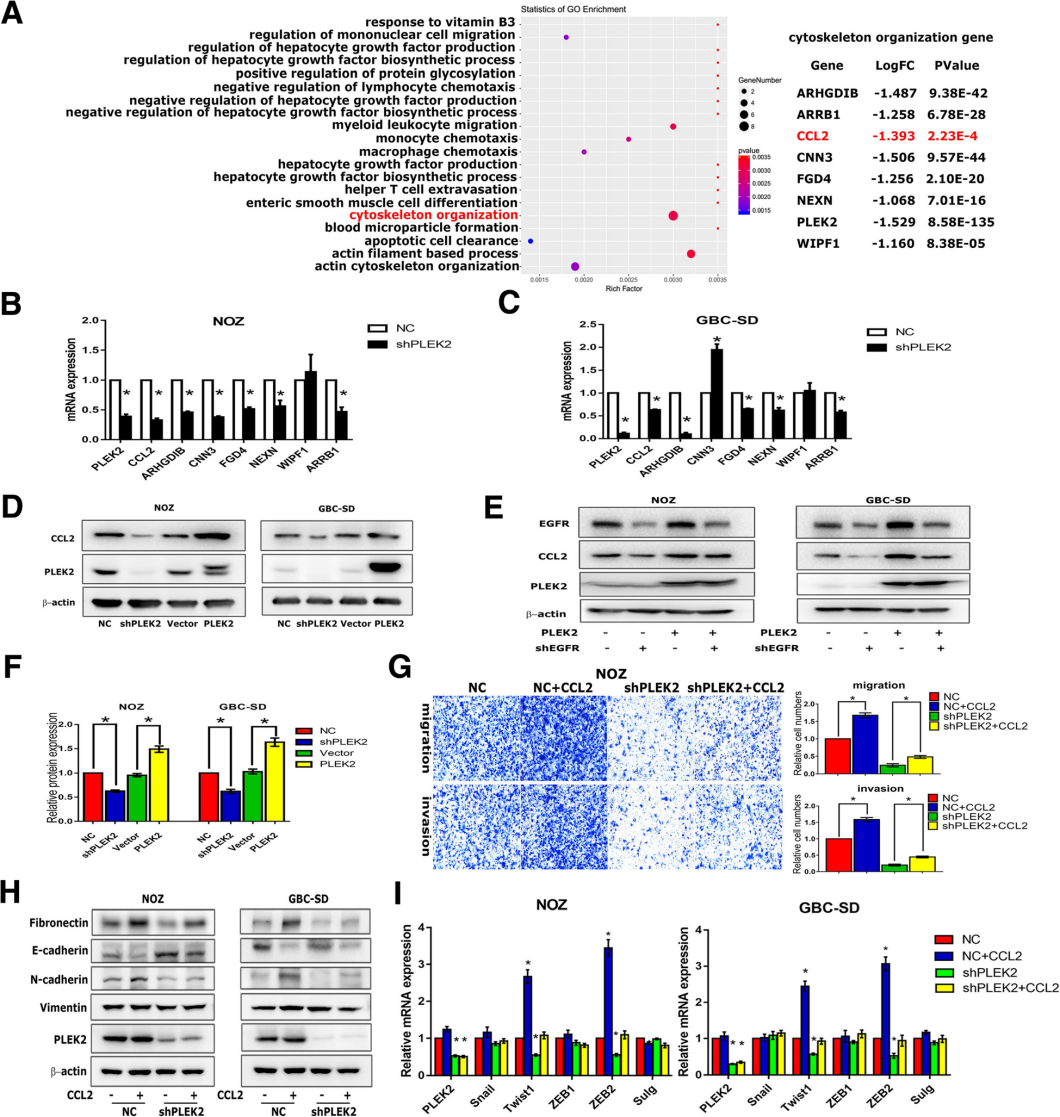
CCL2 was a target gene downstream of PLEK2/EGFR signaling. **a** Differential gene pathway analyses of PLEK2 knockdown cells by mRNA sequencing. **b**, **c** The relative mRNA levels of cytoskeleton organization genes were detected by qRT-PCR. **d** Protein levels of CCL2 in indicated cell lines with either PLEK2 knockdown or overexpression were detected by western blot. **e** Protein levels of CCL2 in PLEK2 overexpression cells with EGFR knockdown were detected by western blot. **f** The protein levels of secreted CCL2 in cellular supernatant were detected by ELISA analysis. **g** Migration and invasion ability of PLEK2 knockdown NOZ cells with 0.1μg/mL CCL2 treatment were measured by transwell. **h** Protein levels of EMT markers in PLEK2 knockdown cells with 0.1μg/mL CCL2 treatment for 24 h were detected by western blot. **i** The relative mRNA levels of EMT related transcription factors in PLEK2 knockdown cells with 0.1μg/mL CCL2 treatment for 24 h were detected by qRT-PCR. All experiments were repeated at least three times, and data were analyzed using student’s t-test. * *P* < 0.05. Error bars indicate SEM

We performed an RNA-sequencing in PLEK2 knockdown cells to identify the downstream targets responsible for the biological function of PLEK2. Consistent with previous studies, the cytoskeleton organization genes were downregulated following PLEK2 knockdown (Fig. 6a). Interestingly, chemokine (C-C motif) ligand 2 (CCL2), which was previously shown to be a target gene of EGFR/STAT3 pathway and promoted migration and invasion in numerous tumors, was also significantly decreased in PLEK2 knockdown cells [24, 25]. qRT-PCR analysis verified PLEK2 regulated cytoskeleton organization genes including CCL2 expression in the mRNA levels (Fig. 6b, c). Western blot analysis verified PLEK2 knockdown markedly reduced CCL2 protein levels, and PLEK2 overexpression increased CCL2 expression (Fig. 6d). The above qRT-PCR and western blot analysis verified CCL2 was a target gene of PLEK2. Meanwhile, silencing EGFR suppressed CCL2 expression in both PLEK2 overexpression and control cells. Above experiments suggests that CCL2 is a target gene of PLEK2/EGFR/STAT3 signaling (Fig. 6e). In consideration of the fact that CCL2 is a secreted protein, we applied enzyme-linked immunosorbent assay (ELISA) to detect the expression of secreted CCL2 in PLEK2 knockdown and overexpression cells. ELISA assay showed PLEK2 knockdown significantly reduced secreted CCL2 protein level, and PLEK2 overexpression remarkably increased secreted CCL2 protein level (Fig. 6f). Then we treated PLEK2 knockdown and control cells with 0.1μg/mL recombinant CCL2 for 24 h to check whether CCL2 could compensate for loss of PLEK2. We found that ectopic CCL2 increased the migration and invasion of both PLEK2 knockdown and control cells (Fig. 6g). Moreover, we found ectopic CCL2 could increase Fibronectin and N-cadherin expression, while decrease E-cadherin expression in both PLEK2 knockdown and control cells (Fig. 6h). In addition, ectopic CCL2 increased transcriptional factors (Twsit1 and ZEB1) expression and rescued the decreased expression of Twsit1 and ZEB1 following PLEK2 knockdown (Fig. 6i). Therefore, these findings indicated that CCL2 was a target gene downstream of PLEK2/EGFR signaling and PLEK2 orchestrated cytoskeleton rearrangement by virtue of CCL2 secretion.

### The prognostic value of combination of PLEK2 and EGFR

**Fig. 7:**
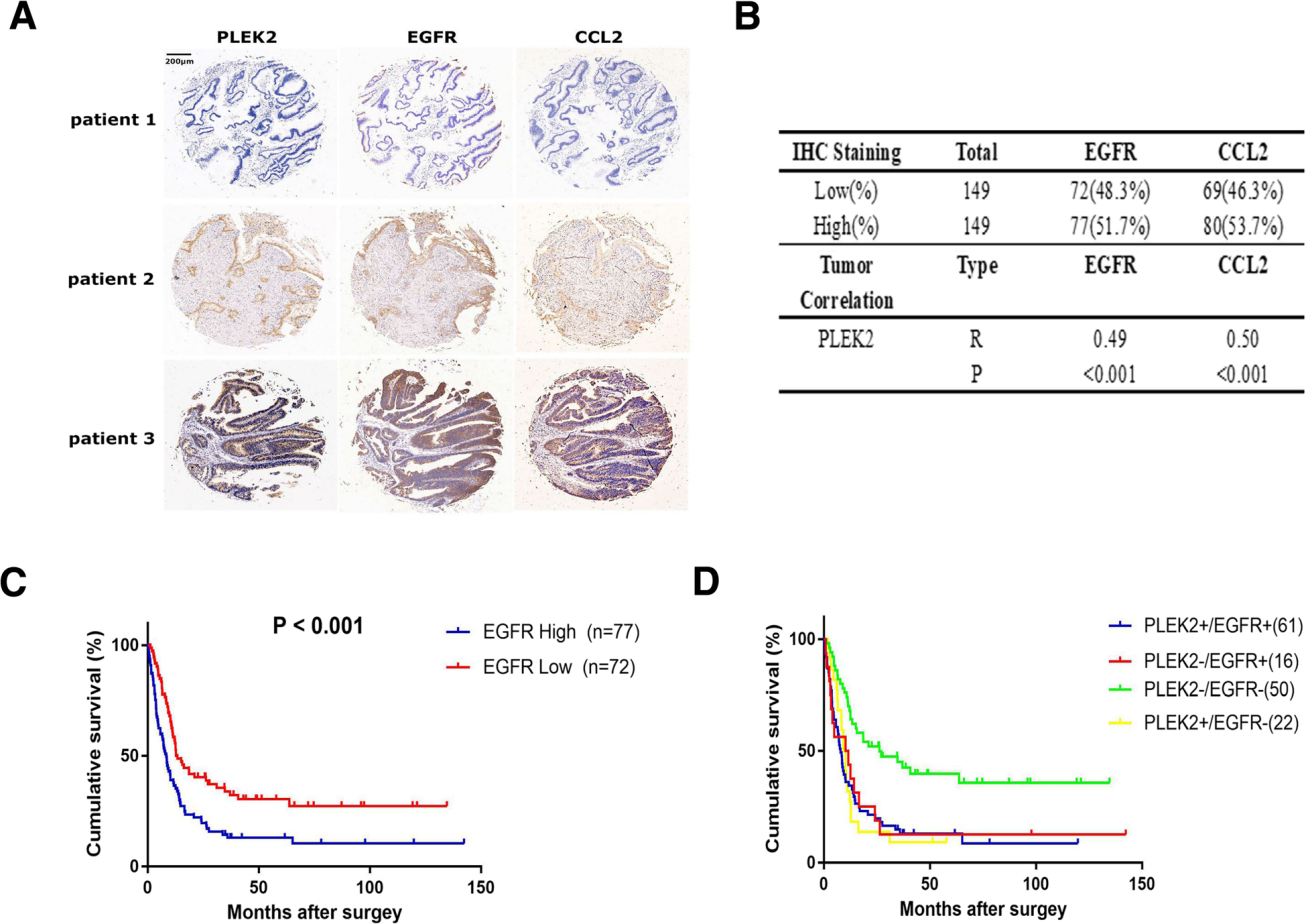
The prognostic value of combination of PLEK2 and EGFR. **a** Representative immunostaining images of PLEK2, EGFR and CCL2 in GBC tissues. **b** Pearson’s correlation analysis between PLEK2 and EGFR/CCL2 in GBC tumour samples. **c** The overall survival for the low and high EGFR expression groups. **d** The overall survival of the combination cohort was stratified by PLEK2 and EGFR expression levels

To evaluate the clinical relevance of PLEK2, EGFR and CCL2, we performed IHC staining assays of the three proteins in gallbladder tissues microarrays including 149 GBC. The results showed the three proteins had similar staining intensity in the certain samples (Fig. 7a). Regression analysis showed a significant positive correlation between the level of PLEK2 and the level of EGFR or CCL2 in GBC tissues (Fig. 7b). Building on the previous data that PLEK2 promoted GBC metastasis and progression through EGFR signaling pathway, we wondered if the combination of PLEK2 and EGFR could be used to better predict GBC survival than either protein. Firstly, we found EGFR high group had a significantly shorter overall survival compared to EGFR low group (HR:1.86, 95%CI:1.29–2.68, *P* < 0.001, Fig. 7c). Importantly, we also found that patients with low expression of both PLEK2 and EGFR had a better prognosis than patients with high expression of either one or two protein (PLEK2+/EGFR+ VS PLEK2−/EGFR-, P < 0.001; PLEK2+/EGFR- VS PLEK2−/EGFR-, P < 0.001; PLEK2−/EGFR+ VS PLEK2−/EGFR-, P < 0.001, PLEK2+ represents PLEK2 high expression group, EGFR+ represents EGFR high expression group, Fig. 7d). Taken together, the combination of PLEK2 and EGFR may serve to predict GBC survival and as a therapeutic target in clinic.

## Discussion

GBC is a malignant tumor with extremely poor prognosis. GBC metastasis is the main cause of GBC-related mortality. However, its mechanism is still poorly understood. To identify the driver genes in GBC metastasis, we performed a mRNA microarray and PLEK2 was selected for further functional studies. In the present study, we demonstrated that PLEK2 was upregulated in GBC tissues. High PLEK2 expression was correlated with high TNM stage and liver metastasis. PLEK2 could also serve as an independent predictor for overall survival of GBC patients. PLEK2 is little known before and its relation with tumor metastasis is poorly understood. A study showed PLEK2 expression was positively correlated with luminal A type breast cancer cells disseminating to bone marrow [11]. Two recent studies demonstrated that PLEK2 could regulate cytoskeleton rearrangement, resulting in large lamellipodia with ruffle formation and cell spreading [9, 10]. As cells move in a motion of lamellipodia was necessary to EMT process, which was regarded as a potent driver conferring cells with metastatic features [26, 27], we investigated whether PLEK2 could promote EMT process and enhance GBC cells migration. As shown in Fig. 2, we found PLEK2 dysregulation modulated EMT markers expression (E-cadherin, N-cadherin and Fibronectin) and remodels cells morphology, resulting in aberrant migration, invasion and metastasis of GBC cells.

EGFR degradation mechanism is not well understood. It is generally thought that EGFR degradation requires for ubiquitination of the receptor, endocytosis of the receptor-ligand complex and finally degradation by both proteasomal and lysosomal hydrolases [28]. EGFR ubiquitination involves the recruitment of ubiquitin ligases like c-CBL or CHIP, to form ligase-receptor complexes [29, 30]. It is widely accepted that the E3 ubiquitin ligase c-CBL-mediated ubiquitination of the receptor is critical for degradation of EGFR [31]. Interestingly, in the present study, we demonstrated that PLEK2 could bind with EGFR and subsequently inhibited EGFR ubiquitination and proteasomal mediated degradation. Moreover, we verified that c-CBL participated in the regulation of EGFR degradation by PLEK2. As shown in Fig. 4, PLEK2 could suppress the binding between c-CBL and EGFR, and the function of PLEK2 overexpression on EGFR was abolished by c-CBL ectopic expression. It has been reported that c-Cbl mainly interacts with EGFR either directly through phosphorylated Tyr1045, or indirectly through Grb2 at Tyr1068 site [28, 32]. But all these binding sites are not in the intracellular tyrosine kinase domain of EGFR. So PLEK2 may not directly compete with c-Cbl for EGFR binding at Tyr1045 or Tyr1068 sites. Regulation of c-CBL activity involves a complex interplay between c-CBL and its many interacting partners. For example, ITSN1 and CIN85 could bind c-CBL to stimulate its activity and enhance EGFR ubiquitylation [33, 34]. However, Spry2 could inhibit the interaction between c-CBL and EGFR by competitive binding with c-CBL [35]. We speculated that PLEK2 might regulate the c-CBL activity by disturbing the interplay between c-CBL and its interacting partners.

One interesting question is how PLEK2 moves to the cell membrane with EGF treatment. Although PLEK2 could bind to PIP2 and PIP3 of cell membrane by its PH domain [9], it is still unknow that how PLEK2 moves to the cell membrane. In this study, we found more PLEK2 protein bound to EGFR after EGF treatment. And interestingly, intracellular tyrosine kinase domain of EGFR is necessary for the interaction between PLEK2 and EGFR. As it’s known that PLEK1 is a major substrate for protein kinase C (PKC) in platelets and leukocytes, and its function in F-actin rearrangement is tightly regulated by PKC-mediated phosphorylation [36]. Just like PLEK1, we presume that EGFR may function as a kinase for PLEK2 and help activate the function of PLEK2. That whether phosphorylation is the necessary step for PLEK2 function and whether EGFR is the major kinase to PLEK2 need further investigation.

The regulation of CCL2 expression by PLEK2 through EGFR/STAT3 signaling is another striking finding of this study. CCL2 is a member of the CC chemokine family which regulates the chemoattraction of macrophages, monocytes, and other inflammatory cells [37]. Recently, CCL2 has been shown to be critical in tumorigenesis and metastasis of numerous solid tumors. CCL2-CCR2 signaling activation enhance metastasis-associated microenvironment and cancer cells interaction, resulting in extravasation, persistent growth of cancer cells and also distant metastasis [24, 38–40]. It is demonstrated that CCL2 induces EMT process dependent on the activation of STAT3 signal and p-STAT3 inhibition suppresses CCL2 expression, leading to reduced invasiveness of tumor cells [41–43]. In this study, we found CCL2 was one of the critical downstream genes of PLEK2. We also demonstrated that PLEK2 regulated EGFR/STAT3 signaling. All these results suggested that PLEK2 regulated CCL2 expression through EGFR/STAT3 signaling. Previous studies indicated CCL2 induced EMT process mainly through the activation of transcription factor Snail [44, 45], but in our study, excretive CCL2 protein could partially rescue the inhibitory effects of PLEK2 knockdown by increasing Twist1 and ZEB1 expressions.

Taken together, PLEK2 activated EGFR/STAT3 signaling, leading to CCL2 transcriptional promotion, and excretive CCL2 enhanced GBC cells migration and invasion in an autocrine form. On the other hand, data of tissue microarray staining demonstrated that PLEK2, EGFR and CCL2 were all activated and correlated with each other in GBC tissues. By analysis the mRNA sequencing data, we found that, in addition to CCL2, PLEK2 modulated the expression of cytoskeleton organization genes, such as ARHGDIB, ARRB1, CNN3 and FGD4, indicating that PLEK2 might promote the metastasis of GBC by orchestrating inflammation network and cytoskeleton organization.

## Conclusions

In summary, we have identified PLEK2 as a key promoter of GBC metastasis, which plays vital roles in EGFR ubiquitination and proteasomal mediated degradation. PLEK2 allows for prolonged activation of EGFR, leading to downstream CCL2 transcriptional overexpression and EMT process activation. The combination of PLEK2 and EGFR may serve to predict GBC survival and as a therapeutic target in clinic.

## Additional files

Additional file 1: **Figure S1A.** Analysis the gene expression differences and its distribution in human cancer cells by bioinformatics data (http://www.broadinstitute.o.rg). (PDF 145 kb)

Additional file 2: **Figure S2A.** Cells construction of PLEK2 down-regulation NOZ and GBC-SD cells (NOZ-shPLEK2, GBC-SD-shPLEK2, respectively), also PLEK2 overexpression NOZ and GBC-SD cells (NOZ-PLEK2, GBC-SD-PLEK2, respectively). (PDF 95 kb)

Additional file 3: **Figure S2B.** Proliferation ability of NOZ and GBC-SD cells with stable PLEK2 knockdown and controls cells were measured by CCK-8 assay. (PDF 91 kb)

Additional file 4 **Figure S2C.** EMT markers of NOZ and GBC-SD cells with stable PLEK2 knockdown and overexpression were detected by qRT-PCR. (PDF 186 kb)

Additional file 5: **Figure S4A.** EGFR expression of PLEK2 overexpression and control cells after 50 ng/ml EGF treatment were detected by IF staining. (PDF 218 kb)

Additional file 6: **Figure S4B.** EGFR mRNA levels of NOZ and GBC-SD cells with stable PLEK2 knockdown and overexpression were detected by qRT-PCR. (PDF 118 kb)

Additional file 7: **Figure S4C.** GBC cells were treated with 100 μM Chloroquine for 8 h, followed by 50 ng/ml EGF stimulation for 5 m. Alterations of EGFR expression in PLEK2 knockdown cells were detected by western blot. (PDF 139 kb)

Additional file 8: **Figure S4D.** Protein levels of EGFR in PLEK2 overexpression cells with increasing ectopic c-CBL expression were detected by western blot. (PDF 93 kb)

Additional file 9: **Figure S5.** Representative images of H&E staining of mouse model. Figure S5A and S5B were the representative images of H&E staining of metastatic focuses in livers, Figure S5C was a representative image of the H&E staining of subcutaneous xenografts. (PDF 1697 kb)

## Data Availability

Please contact the corresponding author for all data requests.

## Abbreviations

CCL2: Chemokine (C-C motif) ligand 2
Co-IP: Co-immunoprecipitation
DEP: Disheveled–Egl-10–pleckstrin
DMSO: Dimethyl sulfoxide
EGFR: Epidermal growth factor receptor
EMT: Epithelial-mesenchymal transition
GBC: Gallbladder cancer
IF: Immunofluorescence
IHC: Immunohistochemistry
OS: Overall survival
PH: Pleckstrin homology
PI3K: Phosphatidylinositol 3-kinase
PLEK2: Pleckstrin-2
TNM stage: Tumor nodule and metastasis stage
