# Supplementary figures and images for: PLEK2 promotes gallbladder cancer invasion and metastasis through EGFR/CCL2 pathway
Source: J Exp Clin Cancer Res. 2019 Jun 10;38:247. doi: 10.1186/s13046-019-1250-8 (PMC6558801; doi:10.1186/s13046-019-1250-8)

mRNA expression (RNAseq): PLEK2

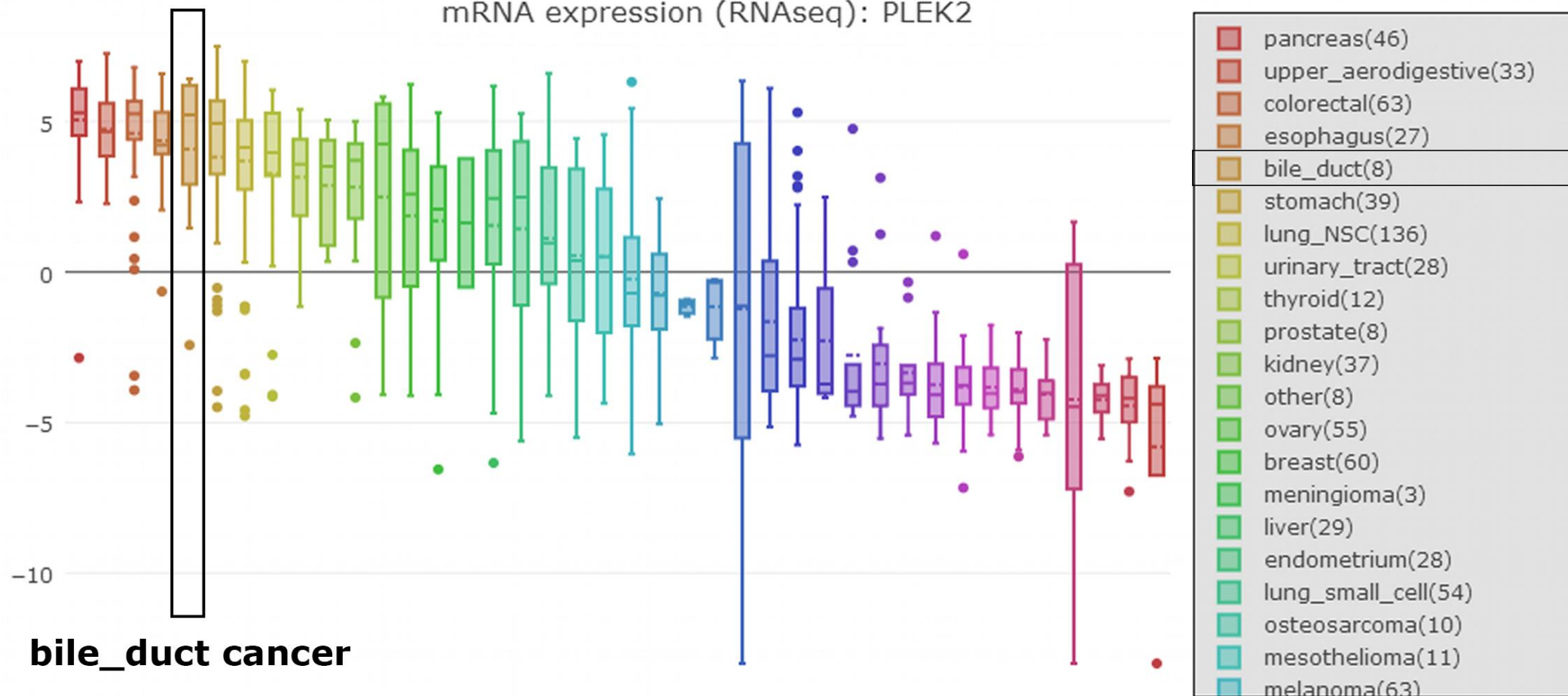

Supplement: Supplementary file 1 — Figure S1A. Analysis the gene expression differences and its distribution in human cancer cells by bioinformatics data (http://www.broadinstitute.o.rg). (PDF 145 kb) [file 13046_2019_1250_MOESM1_ESM.pdf]

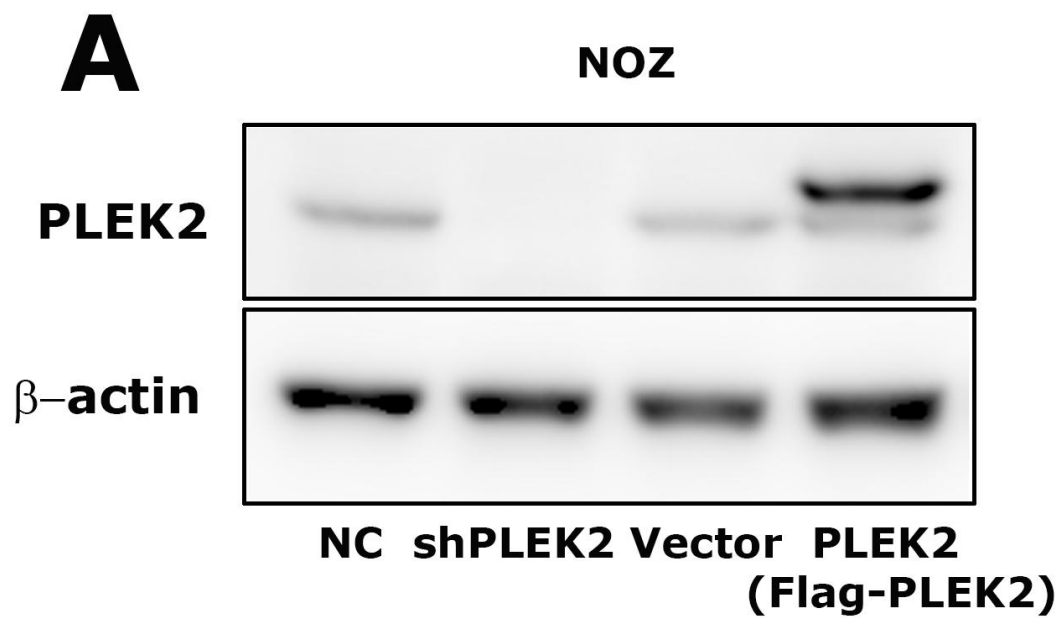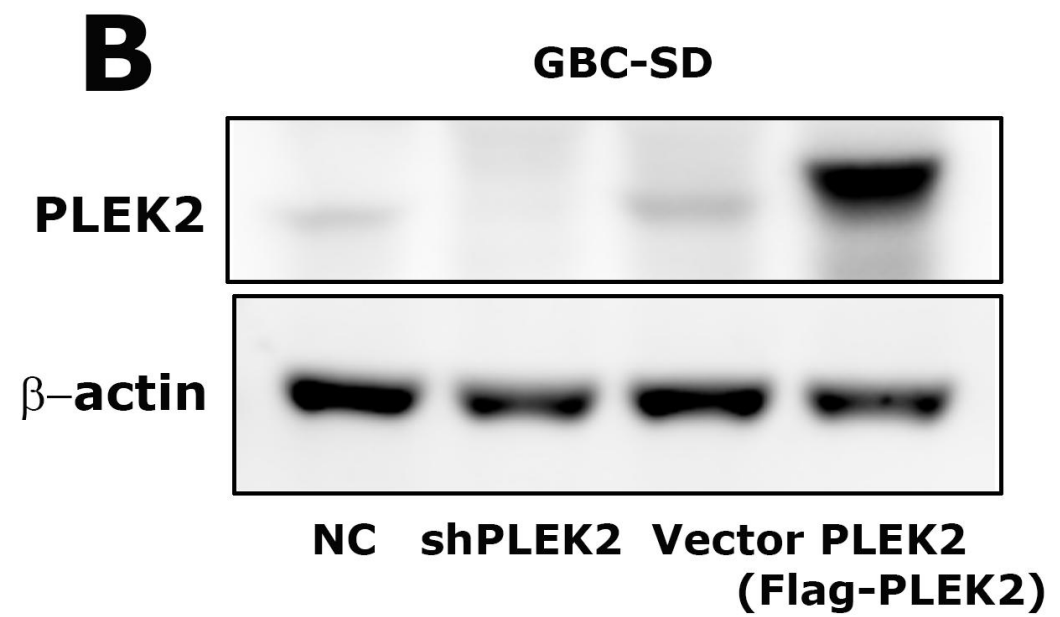

Supplement: Supplementary file 2 — Figure S2A. Cells construction of PLEK2 down-regulation NOZ and GBC-SD cells (NOZ-shPLEK2, GBC-SD-shPLEK2, respectively), also PLEK2 overexpression NOZ and GBC-SD cells (NOZ-PLEK2, GBC-SD-PLEK2, respectively). (PDF 95 kb) [file 13046_2019_1250_MOESM2_ESM.pdf]

**A**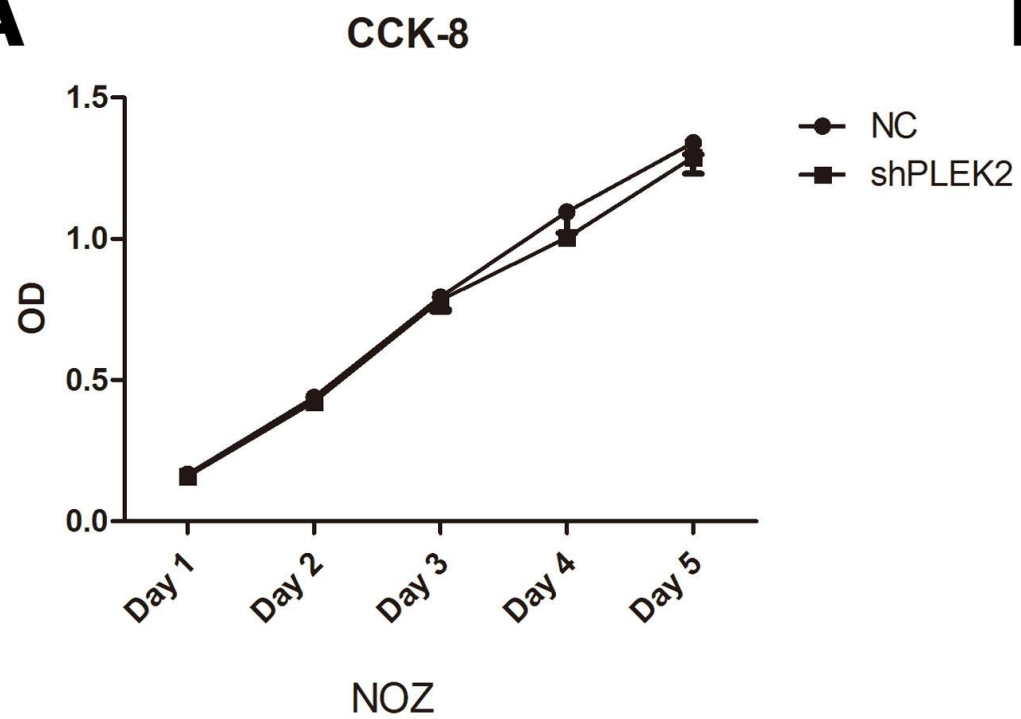**B**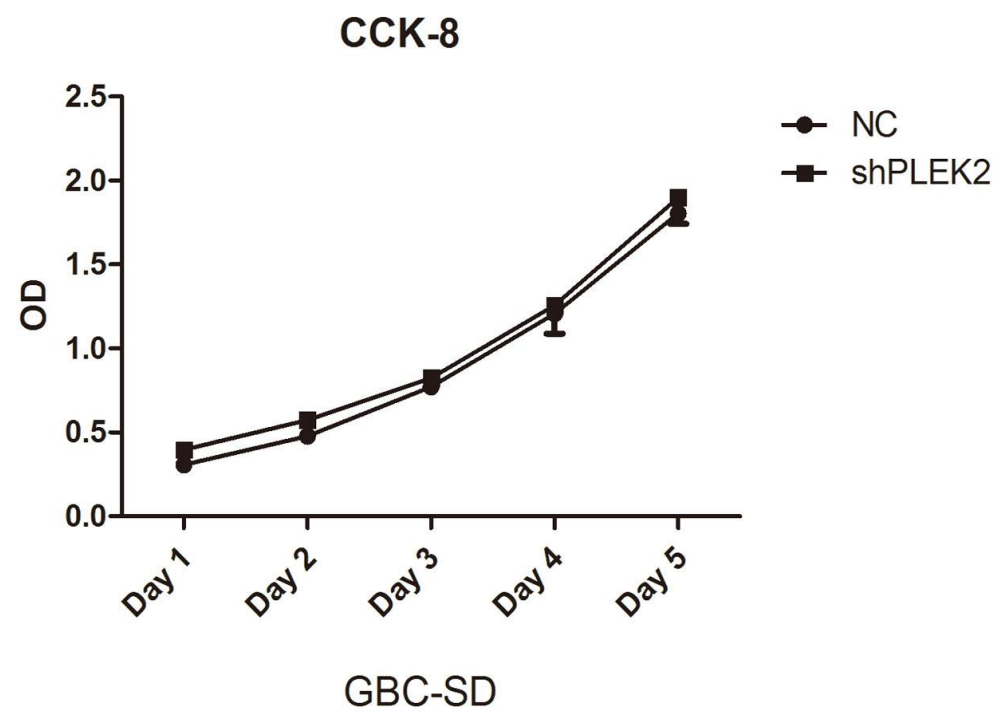

Supplement: Supplementary file 3 — Figure S2B. Proliferation ability of NOZ and GBC-SD cells with stable PLEK2 knockdown and controls cells were measured by CCK-8 assay. (PDF 91 kb) [file 13046_2019_1250_MOESM3_ESM.pdf]

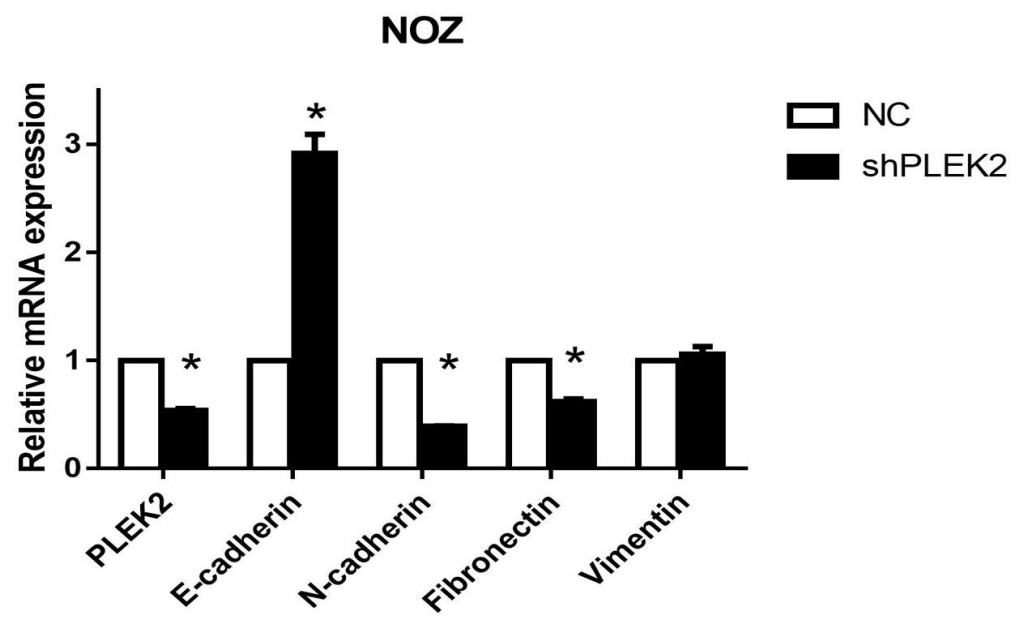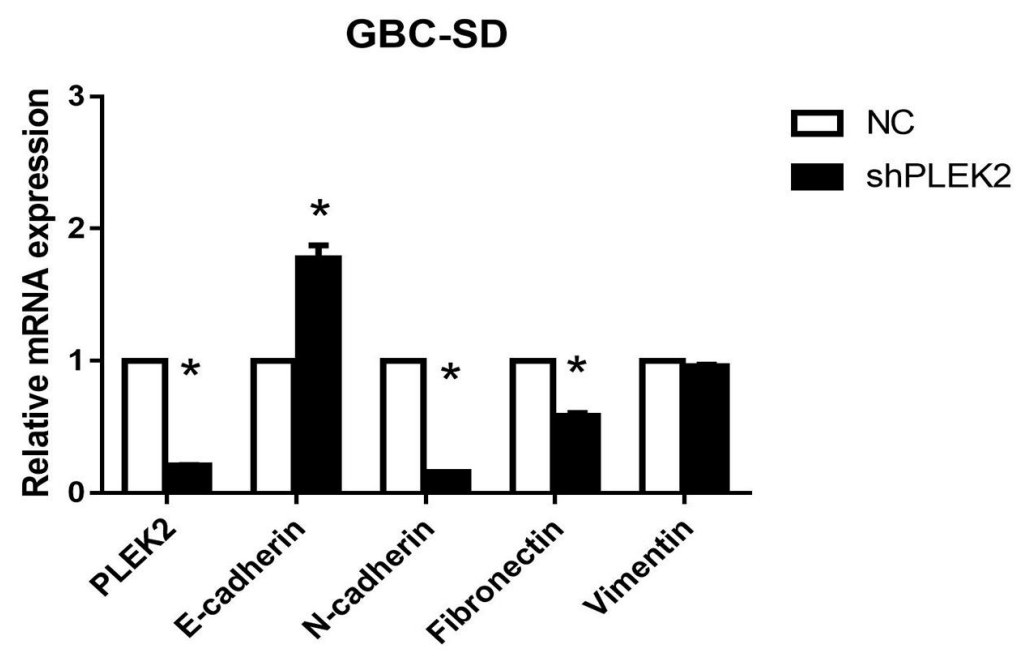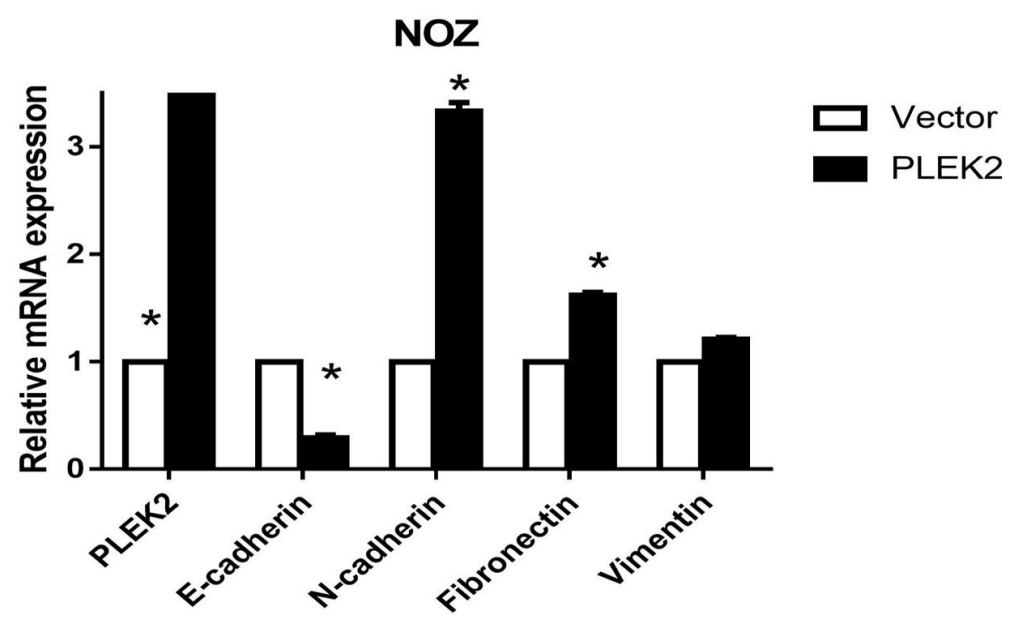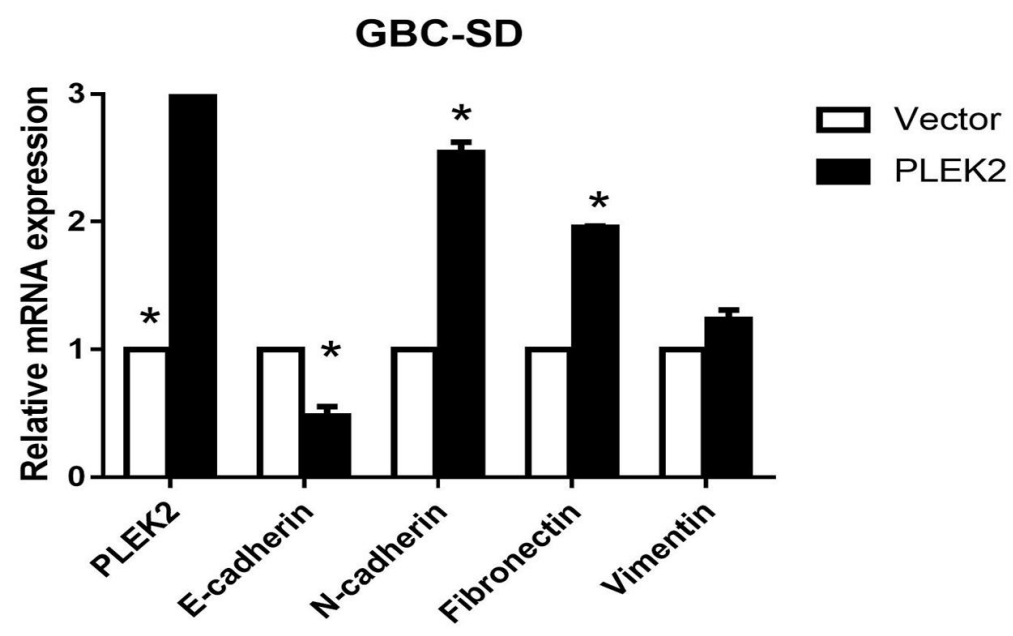

Supplement: Supplementary file 4 — Figure S2C. EMT markers of NOZ and GBC-SD cells with stable PLEK2 knockdown and overexpression were detected by qRT-PCR. (PDF 186 kb) [file 13046_2019_1250_MOESM4_ESM.pdf]

## **GBC-SD**

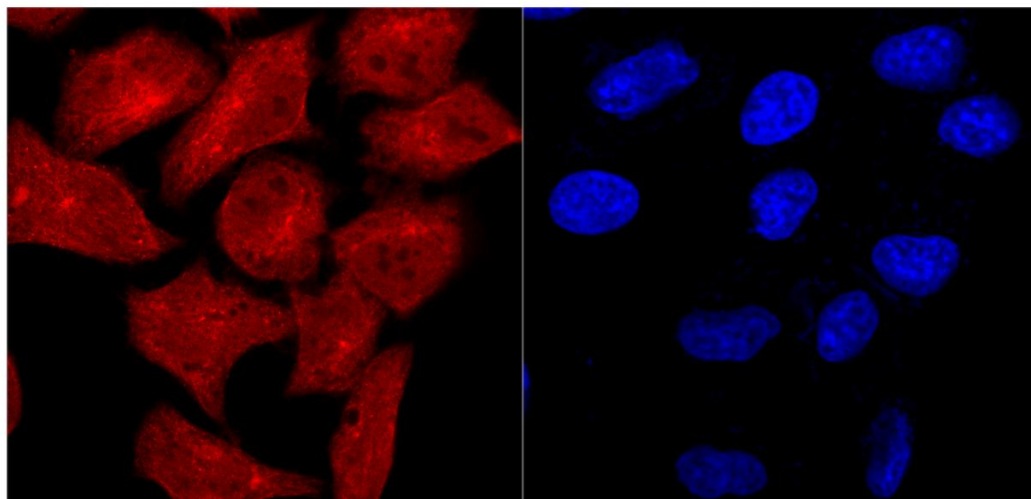

**5min**

**Vector**

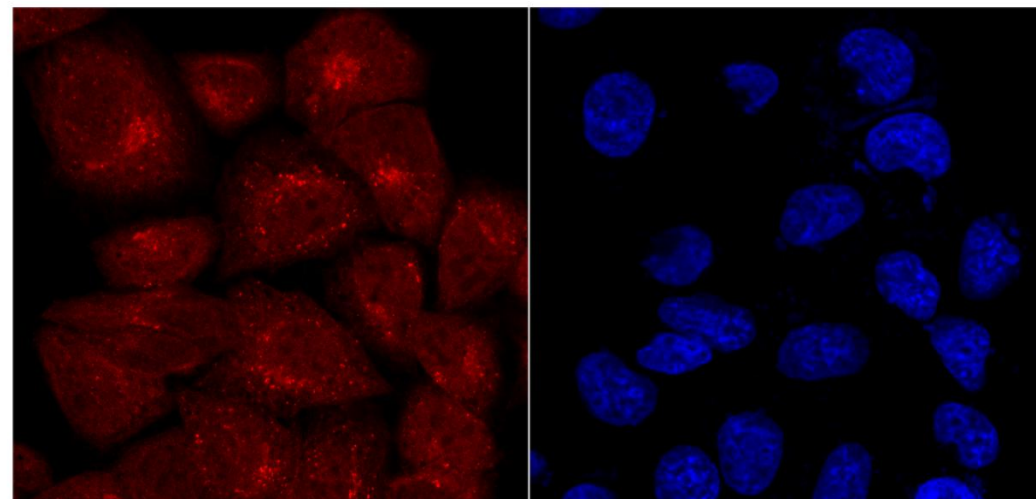

**30min**

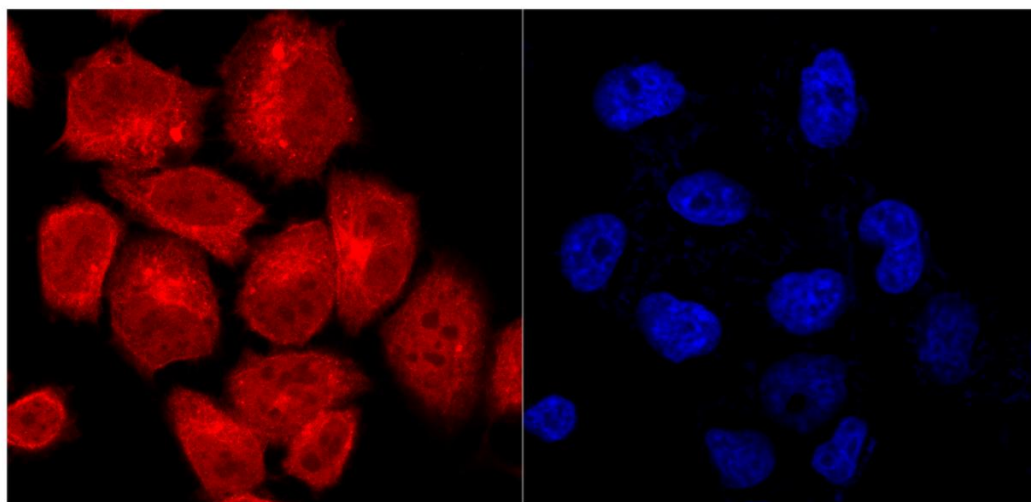

**5min**

**PLEK2**

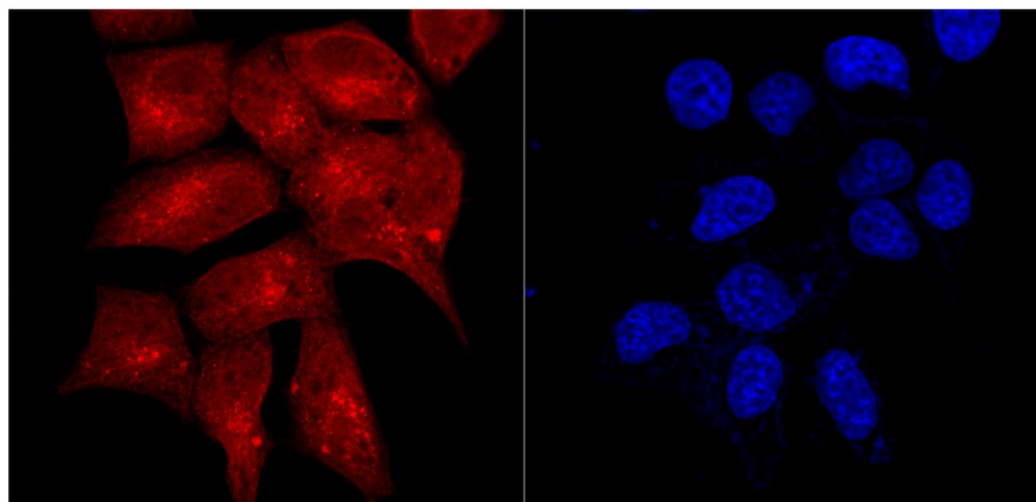

**30min**

Supplement: Supplementary file 5 — Figure S4A. EGFR expression of PLEK2 overexpression and control cells after 50 ng/ml EGF treatment were detected by IF staining. (PDF 218 kb) [file 13046_2019_1250_MOESM5_ESM.pdf]

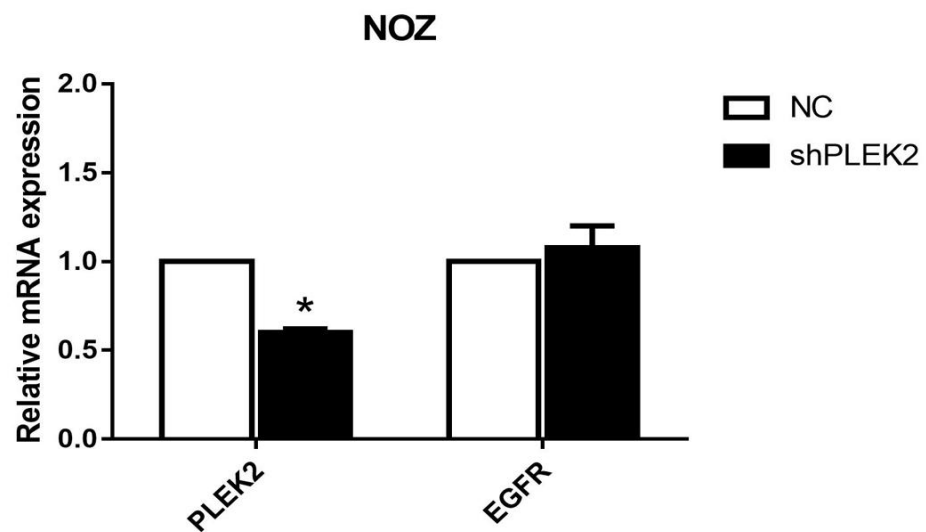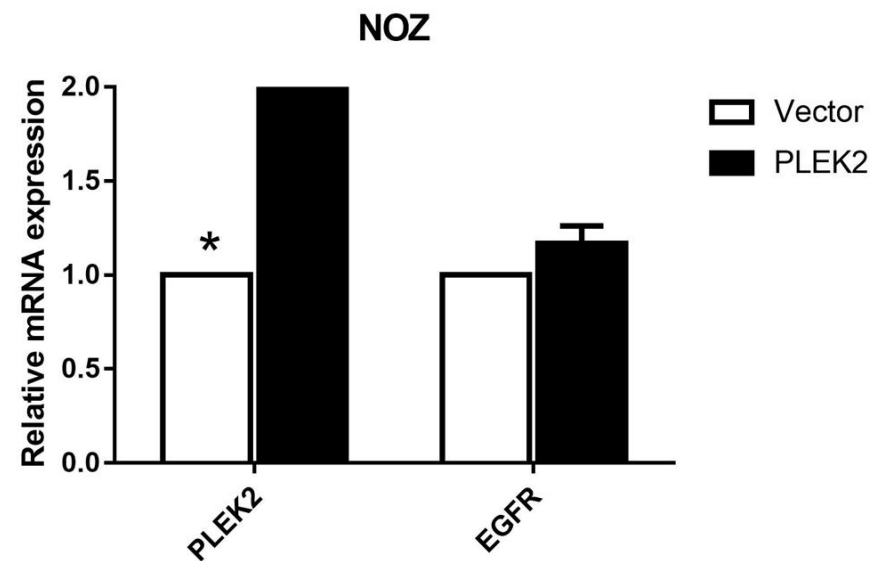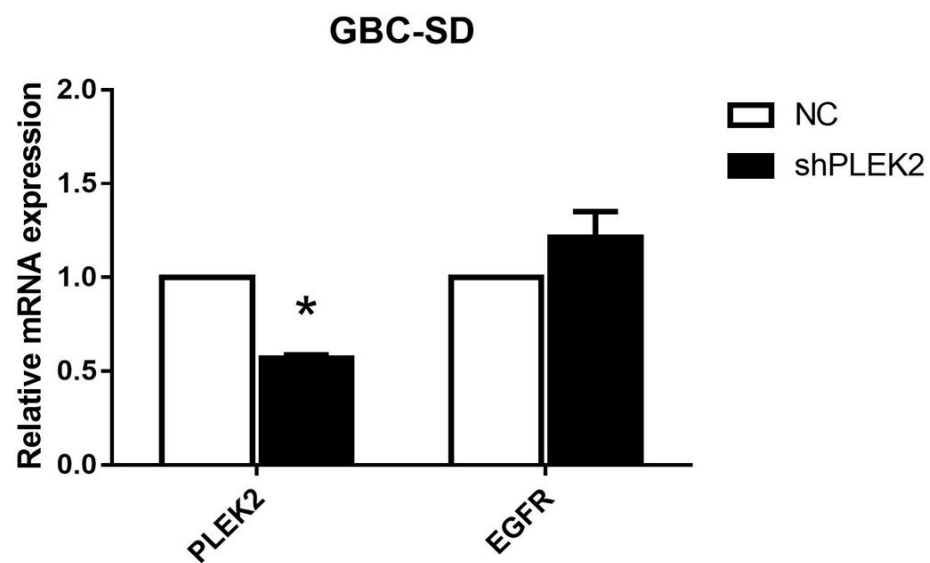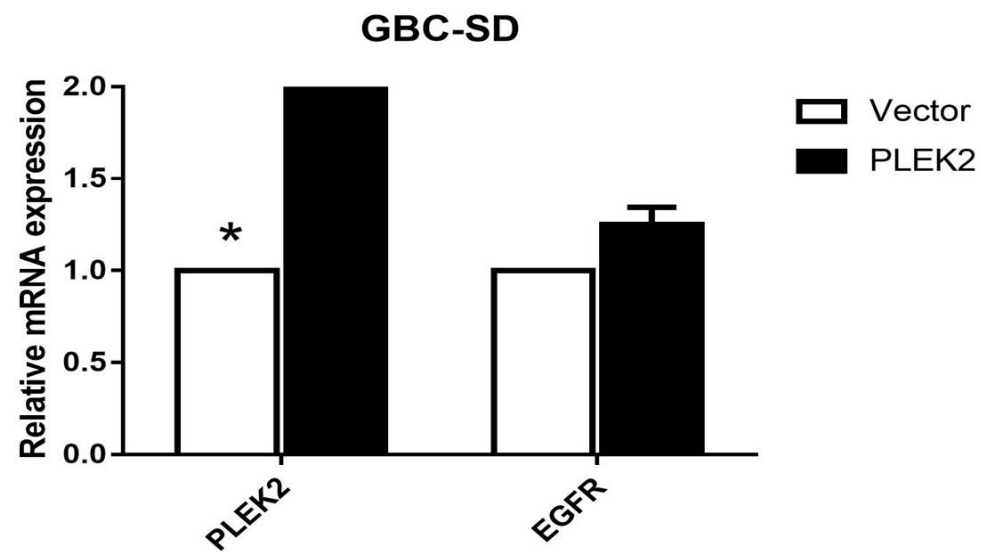

Supplement: Supplementary file 6 — Figure S4B. EGFR mRNA levels of NOZ and GBC-SD cells with stable PLEK2 knockdown and overexpression were detected by qRT-PCR. (PDF 118 kb) [file 13046_2019_1250_MOESM6_ESM.pdf]

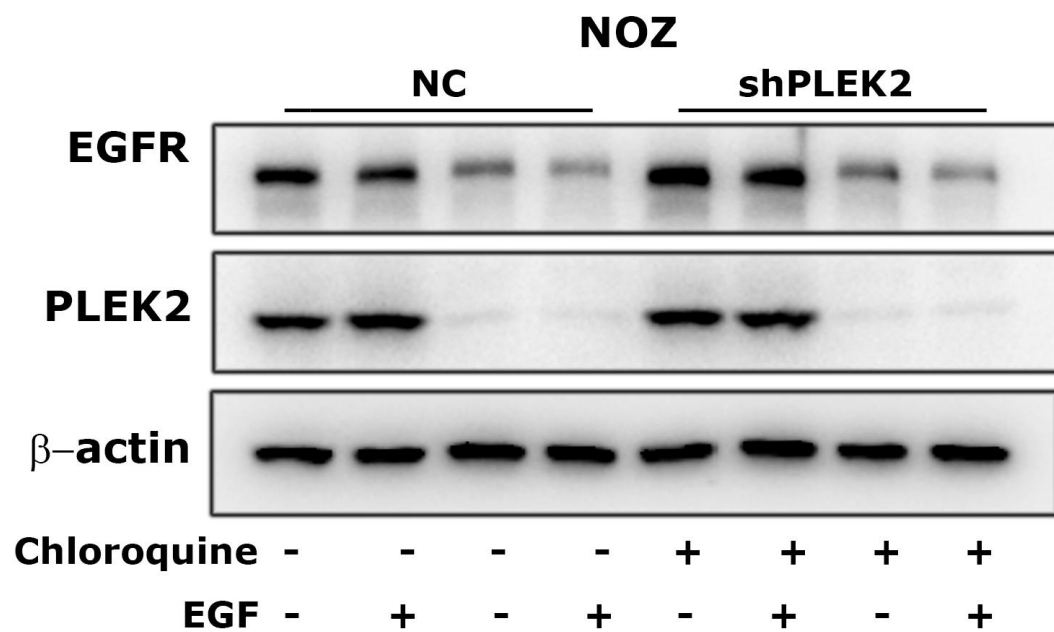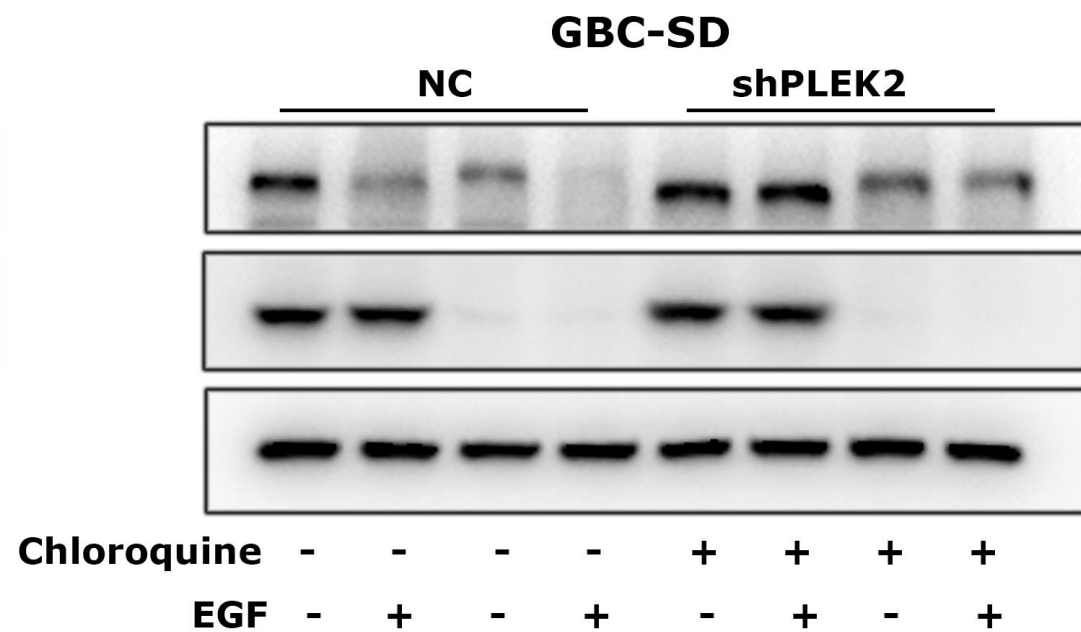

Supplement: Supplementary file 7 — Figure S4C. GBC cells were treated with 100 μM Chloroquine for 8 h, followed by 50 ng/ml EGF stimulation for 5 m. Alterations of EGFR expression in PLEK2 knockdown cells were detected by western blot. (PDF 139 kb) [file 13046_2019_1250_MOESM7_ESM.pdf]

**NOZ**

**EGFR**

**Flag-c-CBL**

**PLEK2**

**$\beta$ -actin**

**Flag-PLEK2**

**Flag-c-CBL**

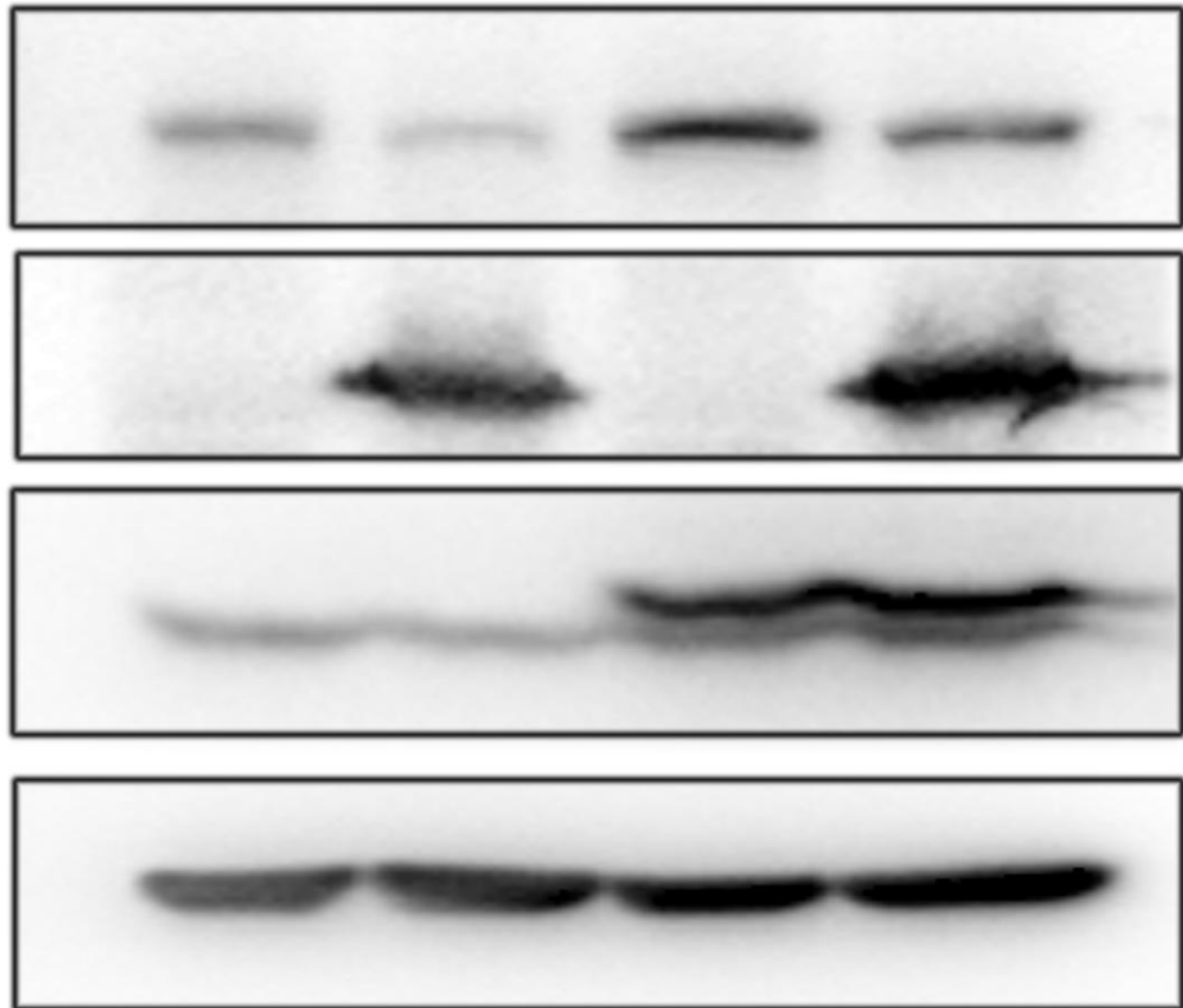

-

-

+

+

-

+

-

+

Supplement: Supplementary file 8 — Figure S4D. Protein levels of EGFR in PLEK2 overexpression cells with increasing ectopic c-CBL expression were detected by western blot. (PDF 93 kb) [file 13046_2019_1250_MOESM8_ESM.pdf]

**A****200X**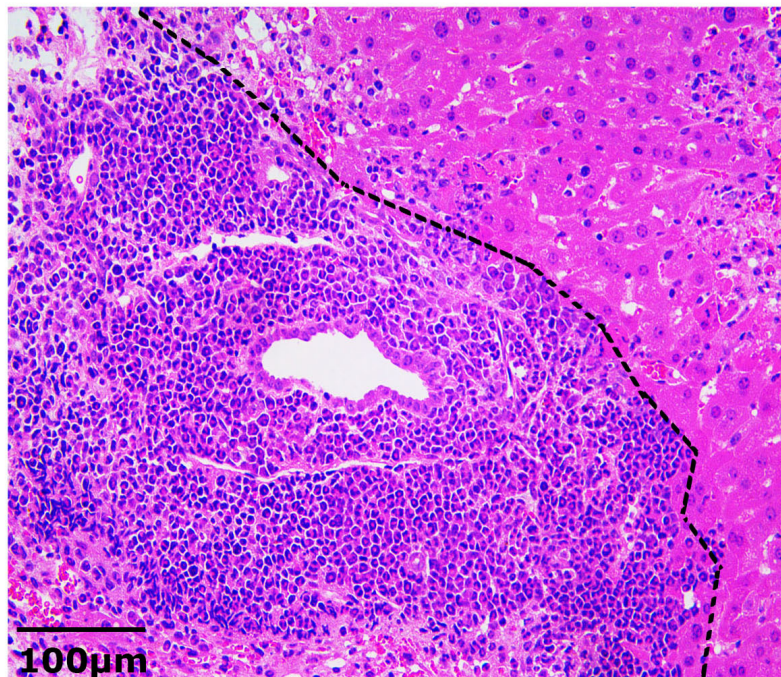**B****200X**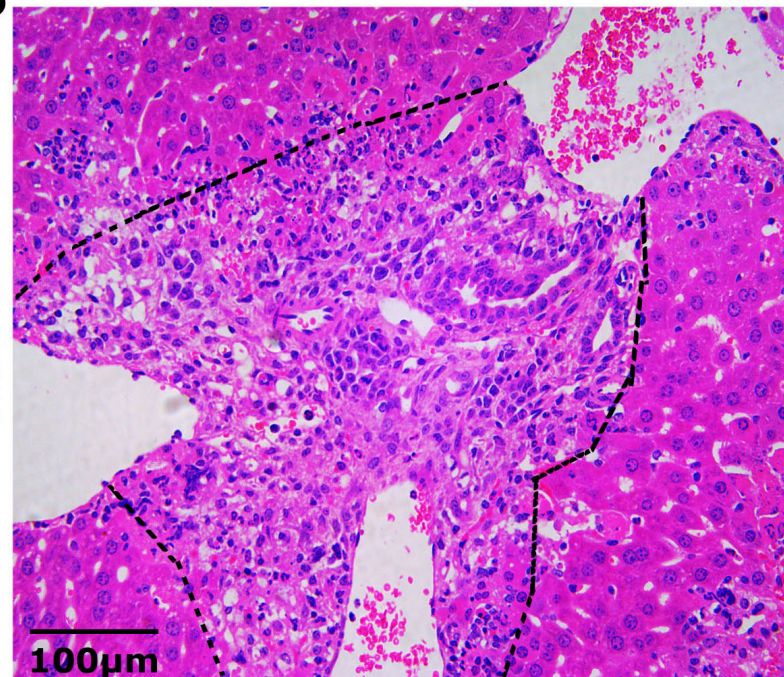**C****200X**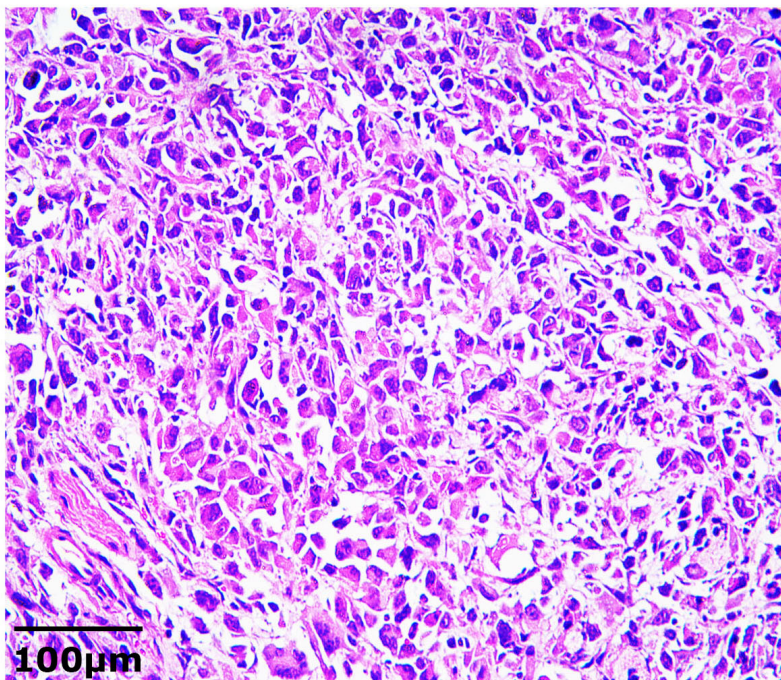

Supplement: Supplementary file 9 — Figure S5. Representative images of H&E staining of mouse model. Figure S5A and S5B were the representative images of H&E staining of metastatic focuses in livers, Figure S5C was a representative image of the H&E staining of subcutaneous xenografts. (PDF 1697 kb) [file 13046_2019_1250_MOESM9_ESM.pdf]
